# Identifying Patient-Recognised Care Practices and Variation in Positive Feedback Content: A Nationwide Cross-Sectional Text Analysis in China

**DOI:** 10.3390/healthcare14203422

**Published:** 2026-10-13

**Authors:** Yazi He, Zijian Cao, Xia Lin, Jingyi Wu, Yetong Zhao, Xixi Yang, Zongjiu Zhang, Hu Chen

**Affiliations:** 1 Institute for Hospital Management, Tsinghua University, Shenzhen 518055, Chinacaozj23@mails.tsinghua.edu.cn (Z.C.);; 2 School of Biomedical Engineering, Tsinghua University, Beijing 100084, China; 3 National Center for Medical Service Administration, National Health Commission of the People’s Republic of China, Beijing 100044, China; 4 Quality Management Department, Sir Run Run Shaw Hospital, School of Medicine, Zhejiang University, Hangzhou 310016, China

**Keywords:** patient feedback, patient experience, free-text comments, text analysis, healthcare quality, hospital management

## Abstract

**Highlights:**

**What are the main findings?**

- Among 115,466 comments retained after screening, general praise accounted for 75.35% and specific positive experiences assigned to care-practice categories for 23.99%; interpersonal interaction and relational care was the most frequently mentioned domain in care-practice comments, especially service attitude and responsibility.
- After covariate adjustment, inpatient comments more often mentioned interpersonal interaction and relational care and less often mentioned care processes and service support than outpatient comments; all age groups of 40 years or older and the higher educational attainment groups more often mentioned clinical and professional care than their respective reference groups.

**What are the implications of the main findings?**

- Distinguishing general praise from specific care practices and analysing feedback in relation to patient composition and care setting can help clinical teams identify care behaviours recognised by different groups and share experience of specific care practices.
- Future research could incorporate information on patient comorbidities, disease severity and previous healthcare use to further explain feedback differences, and use prospective intervention studies to evaluate the effects of categorised feedback and team discussion on patient experience and care processes.

**Abstract:**

**Background/Objectives**: Large-scale evidence linking positive feedback content to patient and care characteristics remains limited. We identified patient-recognised care practices and examined associations with patient, care-setting and hospital characteristics. **Methods**: We conducted a cross-sectional text analysis of 215,690 unique comments from China’s 2024–2025 national patient satisfaction survey across 31 provincial-level regions. Model-assisted screening for positive content covered all output categories. General praise was distinguished from specific positive experiences, with rules and model-assisted judgements supporting multi-label coding into 13 categories across three domains. Two rounds of human coding refined and independently validated the scheme. Multilevel logistic models with hospital random intercepts examined domain mentions with Benjamini–Hochberg false discovery rate (FDR) adjustment. **Results**: Of 115,466 screened comments, 75.35% expressed general praise and 23.99% mentioned care practices (27,698). Independent validation in 600 screened comments yielded a weighted micro-averaged F1 of 95.82% (95% CI 92.63–97.66%) for 13-category coding. Among care-practice comments, interpersonal interaction and relational care was most frequently mentioned (89.84%); service attitude (64.93%) and responsibility (23.49%) were the most frequent categories. Regression included 27,098 comments from 6580 hospitals. Inpatient versus outpatient comments had higher adjusted odds of mentioning interpersonal interaction and relational care (OR 1.23, 95% CI 1.12–1.34) and lower odds of mentioning care processes and service support (OR 0.55, 95% CI 0.49–0.61). Clinical and professional care mentions were positively associated with each age group ≥ 40 versus 20–29 years and each higher education group versus junior secondary or below. These associations remained significant after FDR adjustment in primary and sensitivity analyses. **Conclusions**: Positive feedback should be interpreted in relation to patient composition and care setting. Future studies could incorporate information such as patient comorbidities to explain feedback differences and evaluate the effects of categorised feedback on team learning and service improvement.

## 1. Introduction

Patient experience is an important dimension of healthcare quality and a focus of global efforts to advance people-centred care. The World Health Organization advocates organising health services around people’s needs and preferences [1] and using patient-reported experience to inform service planning and quality improvement [2]. A systematic review of 55 studies found consistent positive associations between better patient experience and clinical safety and effectiveness [3]. Healthcare organisations commonly use structured questionnaires and open-ended comments to understand patient experience, but improvement depends on extracting specific information from feedback and applying it in practice [4,5,6,7,8,9,10,11].

Positive feedback provides specific accounts of care practices recognised by patients. Complaints and negative feedback help identify service failures and safety concerns [12,13], while open-ended comments supplement details that closed-ended questions may not fully capture, such as staff behaviours, care processes and staff–patient interactions [14,15,16,17]. Positive feedback records patients’ praise of service behaviours, professional performance, communication and emotional support. Analysing this content is consistent with learning from excellence, positive deviance and Safety-II approaches, which involve learning from successful care experiences to identify effective practices in everyday care [18,19,20,21].

Previous studies have identified the main content of patient compliments, but less is known about its associations with patient characteristics and care settings. Previous research has examined patients’ evaluations of healthcare in terms of technical and interpersonal aspects [22,23,24]. Analyses of patient letters of compliment have further identified clinical practices, management approaches and interpersonal care behaviours recognised by patients [25]. An Australian study described the distribution of nursing and midwifery compliments across hospital sites and services [26], while a UK study examined the expression and specific content of positive feedback [27]. These studies provide a basis for analysing compliment content, but their findings warrant examination in a broader range of routine survey comments. Because patient characteristics, care settings and hospital characteristics may be interrelated, comparisons of mention rates alone cannot distinguish their associations with feedback content. It is therefore necessary to link comments with patient and hospital information and examine feedback differences across groups and settings while accounting for other factors and the clustering of comments within hospitals.

Analyses of associations between patient characteristics and feedback content were exploratory, without prespecified directional hypotheses. This study aimed to identify specific care practices recognised by patients in routine national survey comments and explore associations between their mention and patient, care-setting and hospital characteristics. Improving patient experience has become a national healthcare priority in China [28], and the national patient satisfaction survey provides a data source for examining these questions. Using 215,690 patient comments from 31 provincial-level regions of China, we identified specific care practices in comments retained after model-assisted screening using an independently validated classification framework comprising three domains and 13 categories. We reported mention rates using screened comments and care-practice comments as separate denominators and used multilevel models to analyse associations of patient characteristics, care setting and hospital characteristics with mentions of the three care domains. Building on previous descriptions of compliment content, we compared differences across groups and settings after covariate adjustment, providing evidence for healthcare organisations to analyse feedback in relation to patient composition and care setting and a reference for similar research in other healthcare systems.

## 2. Materials and Methods

### 2.1. Study Design

We conducted a nationwide cross-sectional text analysis of open-ended comments from a national patient satisfaction survey to identify specific care practices recognised by patients and to examine associations between their mention and patient characteristics, care setting and hospital characteristics. The study involved model-assisted screening, care-practice classification and coding, coding-rule refinement, independent human validation and multilevel regression analysis.

### 2.2. Patient and Public Involvement

Patients and the public were not involved in the design, conduct, reporting or dissemination plans of this research.

### 2.3. Data Source and Comment Selection

Data were drawn from a 2024–2025 national patient satisfaction survey covering hospitals in 31 provincial-level regions of China. The original dataset contained 215,695 open-ended comments. The research team identified and removed five duplicate records using comment identifier, care setting and merged text, leaving 215,690 unique comments, including 100,887 outpatient comments and 114,803 inpatient comments. Information used in the study included patient sex, age group, educational attainment, insurance/payment type and care setting, as well as hospital type, hospital tier, province and an anonymous hospital identifier.

During preliminary data processing, the research team used a customised version of the iFLYTEK Spark large language model (iFLYTEK, Hefei, China) for topic classification and sentiment classification. The model was developed using 4192 patient comments manually annotated by two trained researchers, with disagreements adjudicated by a third expert. The data were divided into training (70%), validation (15%) and test (15%) sets. On the independent test set, F1 scores were 95.55% for sentiment classification, 87.90% for topic classification and 86.03% for joint topic–sentiment classification; performance measures are reported in Supplementary Table S6. The model outputs covered 21 topics and an additional “other” category, with positive, neutral or negative sentiment assigned to the relevant content. A comment was retained for subsequent content classification if it was classified as positive in at least one of the 21 topics or the additional “other” category. All model output categories were included in screening.

In total, model-assisted screening retained 115,466 comments, hereafter termed screened comments; 100,224 comments were not retained.

Screened comments were classified into five main classes according to whether they described a specific positive experience and whether that experience could be assigned to a care-practice category: (1) general praise; (2) specific positive experiences assigned to care-practice categories; (3) positive descriptions not assigned to care-practice categories; (4) factual statements, questions and suggestions; and (5) insufficient information. General praise expressed overall approval without a specific care attribute or behaviour, whereas the second class described a positive experience involving at least one care-practice category. The third class contained 422 comments (0.37% of screened comments). Some did not clearly identify the object of praise or a specific behaviour; others concerned diverse aspects such as financial burden and food preferences. These scattered descriptions did not form clearly defined care-practice categories suitable for comparative analysis, and combining them into one category would have mixed substantively different experiences. We therefore reported their number separately without creating additional care-practice categories or including these comments in the three-domain regression analyses. The fourth class contained interpretable factual statements, questions or suggestions without a specific positive experience; the fifth contained comments whose meaning could not be determined from the original text. Care-practice categories and coding procedures are described in Section 2.4.

**Figure 1:**
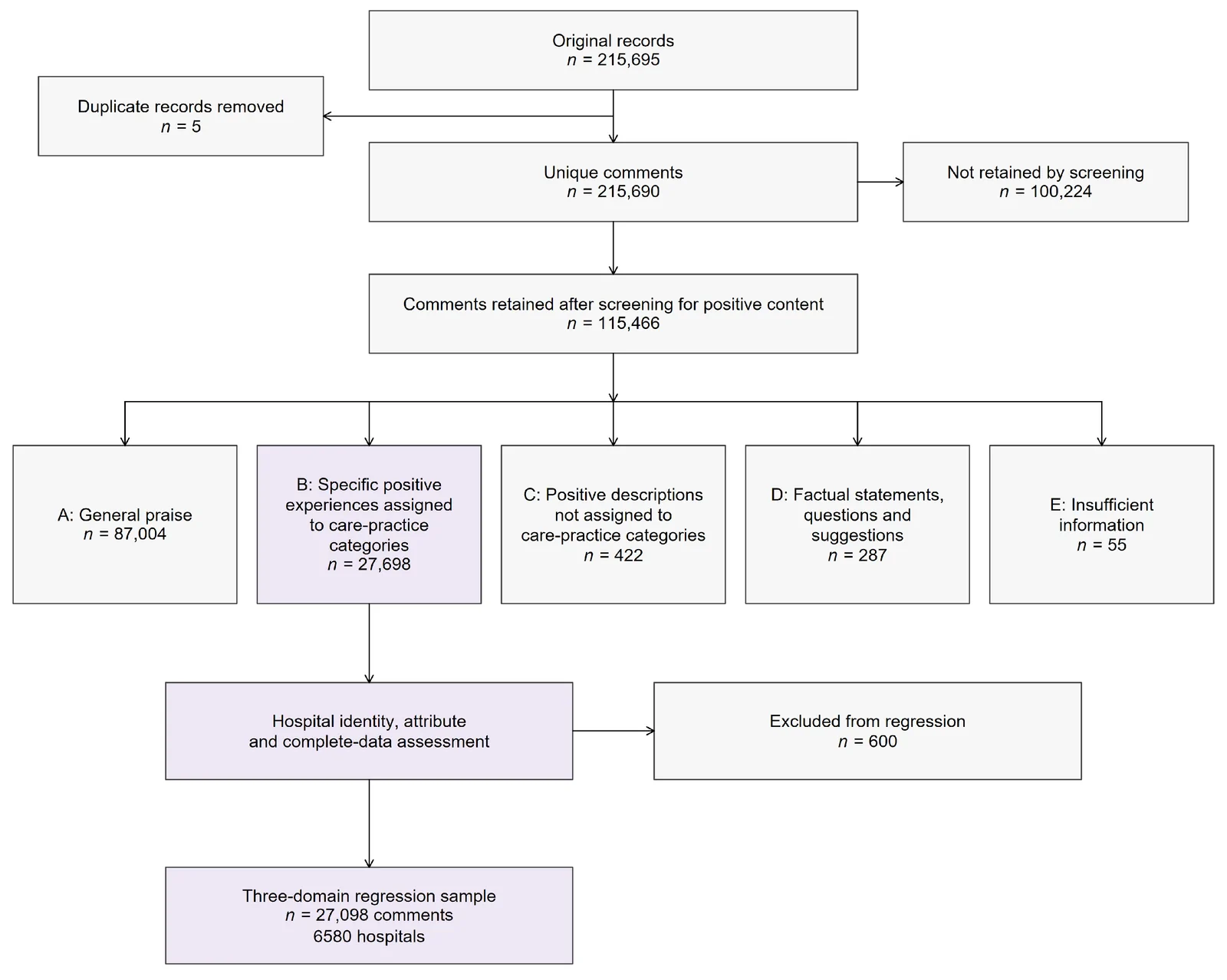
Comment selection and analysis flow. Note: Screened comments were those classified as positive in at least one of the 21 model topics or the additional “other” category. Care-practice comments were assigned to at least one of the 13 care-practice categories. Regression analyses additionally required resolved hospital identity, eligible hospital attributes and complete covariate data. Arrows indicate the flow of comments through screening and analysis; purple shading highlights the pathway from care-practice comments to the regression sample.

After classification, 27,698 comments were assigned to at least one care-practice category, hereafter termed care-practice comments. Regression analyses were restricted to 27,098 care-practice comments from 6580 hospitals after excluding 600 records that did not meet the requirements for hospital identification, hospital attributes and data completeness. The comment selection process is shown in Figure 1.

### 2.4. Care-Practice Classification and Coding

#### 2.4.1. Classification Framework

Previous studies of patient complaints and compliment letters grouped patient feedback into three broad domains: relationships with staff, clinical care and healthcare management [12,25]. We used these domains as the initial framework and adapted the classification to the content and expression of routine survey comments. Research on patient-centred care and patient experience further identifies communication, information provision, emotional support, staff responsiveness, access, coordination and the care environment as important components [29,30], informing the definitions of specific categories and their allocation to domains.

The framework comprised three domains and 13 categories. The first domain was interpersonal interaction and relational care, including service attitude, responsibility, communication or explanation, emotional care and proactive help; these categories describe staff–patient interactions and responses to patients’ needs [12,25,29,30]. The second was clinical and professional care, including professional competence and timely effort, reflecting professional performance and efforts during diagnosis and treatment explicitly praised by patients [22,23,24,25]. The third was care processes and service support, including environmental cleanliness, navigation and guidance, smooth process, one-stop convenience, appointment scheduling and payment and settlement; these categories describe the care environment, service coordination and process arrangements [12,25,29,30].

The research team developed operational definitions, inclusion and exclusion criteria, and illustrative expressions for all 13 categories. Following directed content-analysis principles, category boundaries and decision rules were refined through initial coding and review of disagreements [31]. Category definitions and examples are provided in Supplementary Table S1.

#### 2.4.2. Coding Procedure and Rule Refinement

We used multi-label binary coding: a comment could contain one or more care-practice categories, with each category coded as present or absent. Coding was based on the original comment text, and care-practice labels were assigned only to positive experiences actually described by patients. Topics were not used to infer content that was not explicitly expressed in the original text. For comments containing both positive experiences and criticism or suggestions, positive practice labels supported by the original text were retained; wishes or suggestions were not treated as good practices that had already occurred.

The research team first compiled keywords, common expressions and regular expressions from the comments and used Python (version 3.12.14; pandas version 3.0.1) for text recognition [32,33]. During rule development and refinement, the team used Python to access the Paratera MaaS application programming interface (API) and call DeepSeek-R1 to assist with comments awaiting classification and targeted checks. The model received the original comment text, category definitions and decision criteria, and returned classifications with supporting text excerpts. Researchers reviewed model judgments alongside human coding to identify expressions missed by the rules, clarify category boundaries and refine inclusion and exclusion criteria. Recognition rules were added for expressions that met existing category definitions. Model classifications checked against the original text and rule-based results were combined to produce the final coding. Python was used to consolidate category labels and check that label combinations complied with the decision criteria. The arrangements for human coding in rule refinement and independent validation are described in Section 2.4.3.

#### 2.4.3. Coding Refinement and Independent Human Validation

Two rounds of human coding were conducted. In the first round, two coders independently coded 1100 comments using the preliminary 13-category framework. Disagreements were discussed to refine category definitions and coding rules. This round was used for development of the coding scheme.

After the coding scheme had been refined, we selected a stratified evaluation sample of 600 comments from the screened comments: 150 general-praise comments, 250 comments assigned to care-practice categories, 80 positive descriptions not assigned to care-practice categories, 65 comments containing factual statements, questions or suggestions, and 55 comments with insufficient information. The 250 care-practice comments were further stratified by rare labels to improve coverage of low-frequency categories. For independent human validation, two coders independently classified all 600 comments without access to the program classifications. After independent coding, disagreements between the two coders were resolved through discussion to establish the human reference standard independently of the program classifications. Program classifications were then compared with this reference standard to evaluate coding performance.

Percentage agreement and Cohen’s kappa were calculated from the independent human classifications before discussion [34,35]. The program classifications were compared with the reviewed human reference standard. For each of the five main classes and 13 care-practice categories, we calculated positive predictive value (precision), sensitivity (recall) and F1 score. Micro-averaged and macro-averaged F1 scores were also calculated for the 13-label classification. Estimates were weighted by the ratio of the population size to the sample size in each sampling stratum and reported with 95% confidence intervals. Unweighted results and the number of comments classified as positive by the human reference standard were also reported. Confidence interval estimation accounted for the stratified sampling design; intervals that could not be reliably estimated were marked as not estimable and explained in the Supplementary Tables.

### 2.5. Variables

The three domains and 13 categories were used to describe the content of care-practice comments.

We constructed three binary regression outcomes corresponding to interpersonal interaction and relational care, clinical and professional care, and care processes and service support. Each outcome was coded as 1 if a comment mentioned at least one constituent category and 0 otherwise, without weighting individual categories. The domains were not mutually exclusive.

Covariates included patient sex, age group, educational attainment, insurance/payment type, care setting, hospital tier, hospital type and comment length, with province fixed effects also included. These variables were used to analyse associations of patient and care characteristics with comment content; comment length accounted for differences in the amount of text available for identifying care practices. Care setting was classified as outpatient or inpatient, and hospital tier as non-tertiary or tertiary, with non-tertiary hospitals comprising primary and secondary hospitals.

### 2.6. Statistical Analysis

We first used descriptive statistics to summarise the five main classes of screened comments and the three domains and 13 categories in care-practice comments. Domain and category percentages were calculated using both the 115,466 screened comments and the 27,698 care-practice comments as denominators. With multi-label coding, the sum of percentages across categories or domains could exceed 100%.

In the 27,098-comment regression sample, we then described the percentages mentioning each of the 13 categories by outpatient versus inpatient setting and non-tertiary versus tertiary hospital tier. Each percentage used the total number of comments in the corresponding group as its denominator. These comparisons were descriptive, without category-level significance testing; complete results are provided in Supplementary Table S4.

We fitted separate two-level binary logistic regression models for the three care domains, with hospital random intercepts to account for the clustering of comments within hospitals. Each model included the covariates specified in Section 2.5 and province fixed effects. Results were reported as adjusted odds ratios (ORs) with 95% confidence intervals (CIs). Conventional 95% CIs described the precision of individual association estimates, while the Benjamini–Hochberg method was used for false discovery rate (FDR) adjustment of multiple comparisons. Statistical significance was defined as an adjusted *p* value < 0.05. In the primary analysis, all 150 estimable non-intercept fixed-effect coefficients across the three models, including province fixed-effect coefficients, were included in a single FDR adjustment family.

Sensitivity analyses examined both sample restrictions and model specification. Analyses were repeated after restricting the sample to secondary and tertiary hospitals, and single-level logistic regression with hospital-clustered robust standard errors was fitted in both the full regression sample and the restricted sample. Covariates were unchanged. For each combination of sample and model specification, FDR adjustment jointly covered all estimable non-intercept fixed-effect coefficients across the three domain models as a separate testing family. We compared association directions, effect estimates and FDR-adjusted statistical significance to assess sensitivity to sample restrictions and model specification. Complete regression and sensitivity analysis results are provided in Supplementary Table S5.

Data processing, text coding and model API calls were conducted in Python 3.12.14 (with pandas 3.0.1); regression analyses were conducted in Stata/MP 19; and FDR results were verified and figures produced in R 4.4.1.

## 3. Results

### 3.1. Analytic Samples

Characteristics of the full comment sample, care-practice comments and the regression sample are shown in Table 1. The original dataset contained 215,695 comments. After five duplicate records were removed, the full sample included 215,690 comments, comprising 100,887 outpatient comments and 114,803 inpatient comments. Model-assisted screening retained 115,466 comments classified as positive in at least one of the 21 topics or the additional “other” category.

General praise accounted for 75.35% (*n* = 87,004) of screened comments, and specific positive experiences assigned to care-practice categories accounted for 23.99% (*n* = 27,698). The remaining comments included 422 positive descriptions not assigned to care-practice categories (0.37%), 287 comments containing factual statements, questions or suggestions (0.25%), and 55 comments with insufficient information (0.05%). The comment selection process is shown in Figure 1.

Of the 27,698 care-practice comments, 600 did not meet the requirements for hospital identification, hospital attributes and data completeness. The regression sample comprised 27,098 comments from 6580 hospitals, including 11,336 outpatient and 15,762 inpatient comments, and 14,160 comments from tertiary hospitals and 12,938 from non-tertiary hospitals.

### 3.2. Human Coding Agreement and Classification Performance

In the second round of independent human validation involving 600 comments, agreement between the two coders on the five main classes before discussion was 91.33%, with a Cohen’s kappa of 0.880. After weighting by sampling stratum, agreement was 97.21% and kappa was 0.927.

Across the 13 care-practice categories, unweighted agreement ranged from 98.00% to 99.83%, and category-specific Cohen’s kappa values ranged from 0.831 to 0.990. Supplementary Table S2 reports category-specific human coding agreement.

Using the human coding established through discussion and review as the reference, the program identified comments mentioning at least one care-practice category with a weighted positive predictive value of 97.09%, sensitivity of 97.84% and F1 score of 97.46% (95% CI 93.22–99.07%). The weighted micro-averaged F1 score for 13-label classification was 95.82% (95% CI 92.63–97.66%), and the macro-averaged F1 score was 93.87% (95% CI 90.33–96.17%).

Weighted F1 scores across the 13 care-practice categories ranged from 83.06% to 100.00%. Timely effort and navigation and guidance had relatively lower F1 scores, at 83.06% and 84.13%, respectively. Category-specific positive predictive values, sensitivities, F1 scores and human-positive counts are reported in Supplementary Table S3.

### 3.3. Patient-Recognised Care Practices

Among all 115,466 screened comments, general praise was the most common class (75.35%), followed by comments assigned to at least one care-practice category (23.99%). Using all screened comments as the denominator, interpersonal interaction and relational care, clinical and professional care, and care processes and service support were mentioned in 21.55%, 4.06% and 2.07%, respectively. Among the 27,698 care-practice comments, the corresponding proportions were 89.84% (*n* = 24,883), 16.91% (*n* = 4684) and 8.64% (*n* = 2392) (Table 2).

Using the 27,698 care-practice comments as the denominator, service attitude and responsibility were the most frequently mentioned categories, accounting for 64.93% (*n* = 17,984) and 23.49% (*n* = 6507), respectively. Within interpersonal interaction and relational care, communication or explanation, emotional care and proactive help were mentioned in 13.04% (*n* = 3612), 9.23% (*n* = 2556) and 2.67% (*n* = 739) of comments, respectively. Clinical and professional care primarily involved professional competence (14.53%, *n* = 4024); timely effort was mentioned in 2.75% (*n* = 761).

Within care processes and service support, environmental cleanliness, smooth process, and navigation and guidance were mentioned in 4.39% (*n* = 1216), 2.90% (*n* = 804) and 1.33% (*n* = 367) of comments, respectively. One-stop convenience, appointment scheduling, and payment and settlement were mentioned infrequently, in 29, 17 and 34 comments, respectively. A comment could mention more than one category or domain, so percentages across categories and domains could exceed 100%.

### 3.4. Differences by Care Setting and Hospital Tier

**Figure 2:**
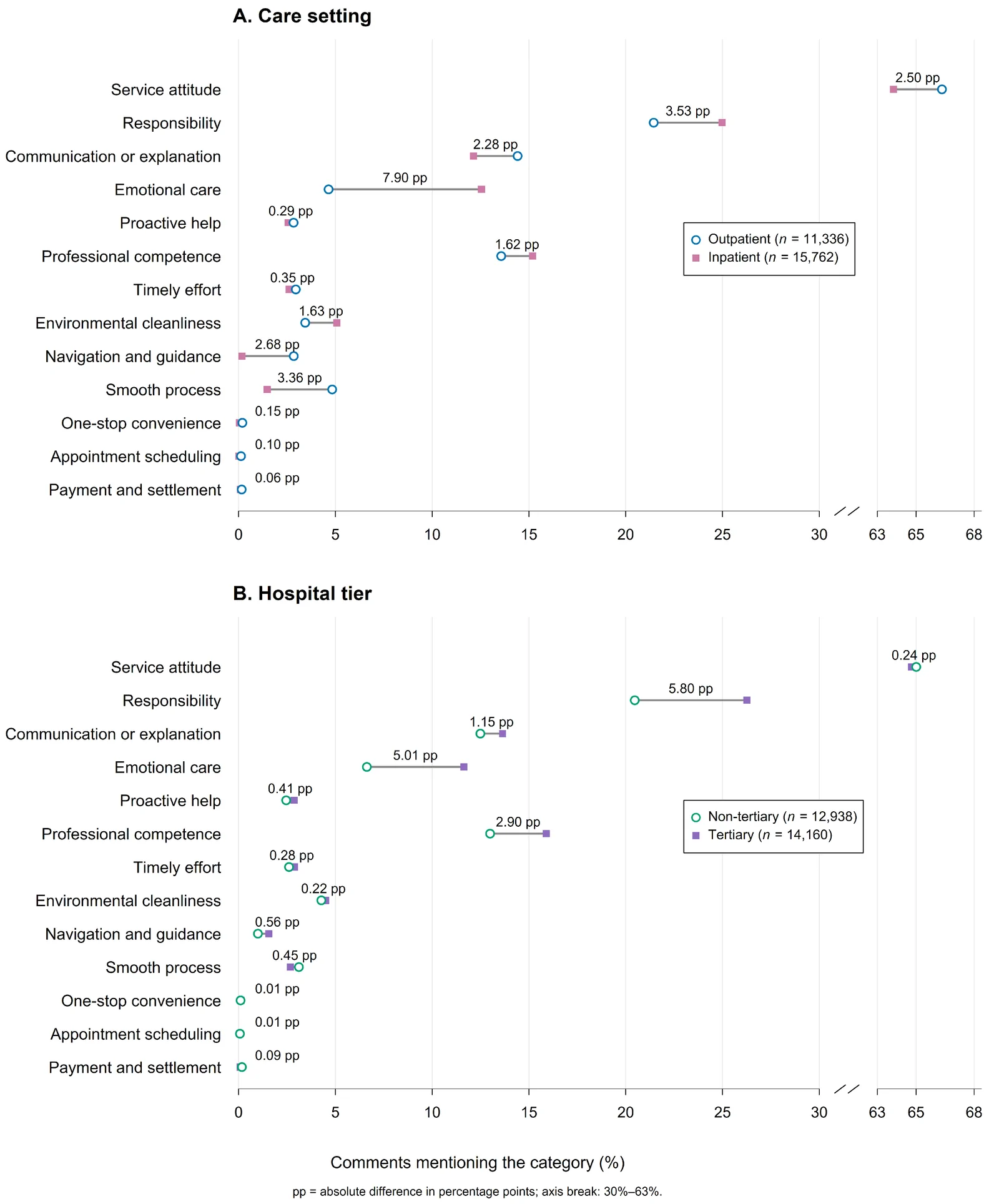
Mention rates for 13 care-practice categories by care setting and hospital tier. Note: The figure shows unadjusted descriptive comparisons in the regression sample of 27,098 comments. Panel (**A**) compares outpatient comments (blue open circles; *n* = 11,336) with inpatient comments (pink squares; *n* = 15,762). Panel (**B**) compares non-tertiary hospital comments (green open circles; *n* = 12,938) with tertiary hospital comments (purple squares; *n* = 14,160). Non-tertiary hospitals include primary and secondary hospitals. Horizontal lines connect the group-specific mention rates, and labels indicate the absolute differences in percentage points (pp), calculated from unrounded rates. The horizontal axis omits the interval between 30% and 63%; both retained intervals use the same scale. Categories may overlap. No category-level significance tests were performed. Complete counts and percentages are provided in Supplementary Table S4.

In the 27,098-comment regression sample, outpatient comments more often mentioned service attitude (66.34% vs. 63.84%), communication or explanation (14.41% vs. 12.14%), navigation and guidance (2.85% vs. 0.17%), and smooth process (4.83% vs. 1.48%) than inpatient comments. Inpatient comments more often mentioned responsibility (24.97% vs. 21.44%), emotional care (12.55% vs. 4.65%), professional competence (15.19% vs. 13.57%), and environmental cleanliness (5.07% vs. 3.44%) than outpatient comments (Figure 2A).

Comments from tertiary hospitals more often mentioned responsibility (26.26% vs. 20.47%), emotional care (11.64% vs. 6.62%), and professional competence (15.90% vs. 12.99%) than those from non-tertiary hospitals. Service attitude was mentioned at similar rates in tertiary and non-tertiary hospitals (64.77% vs. 65.01%; Figure 2B). Underlying counts and full subgroup results for all 13 categories are provided in Supplementary Table S4.

### 3.5. Adjusted Associations with Mentions of the Three Care Domains

**Figure 3:**
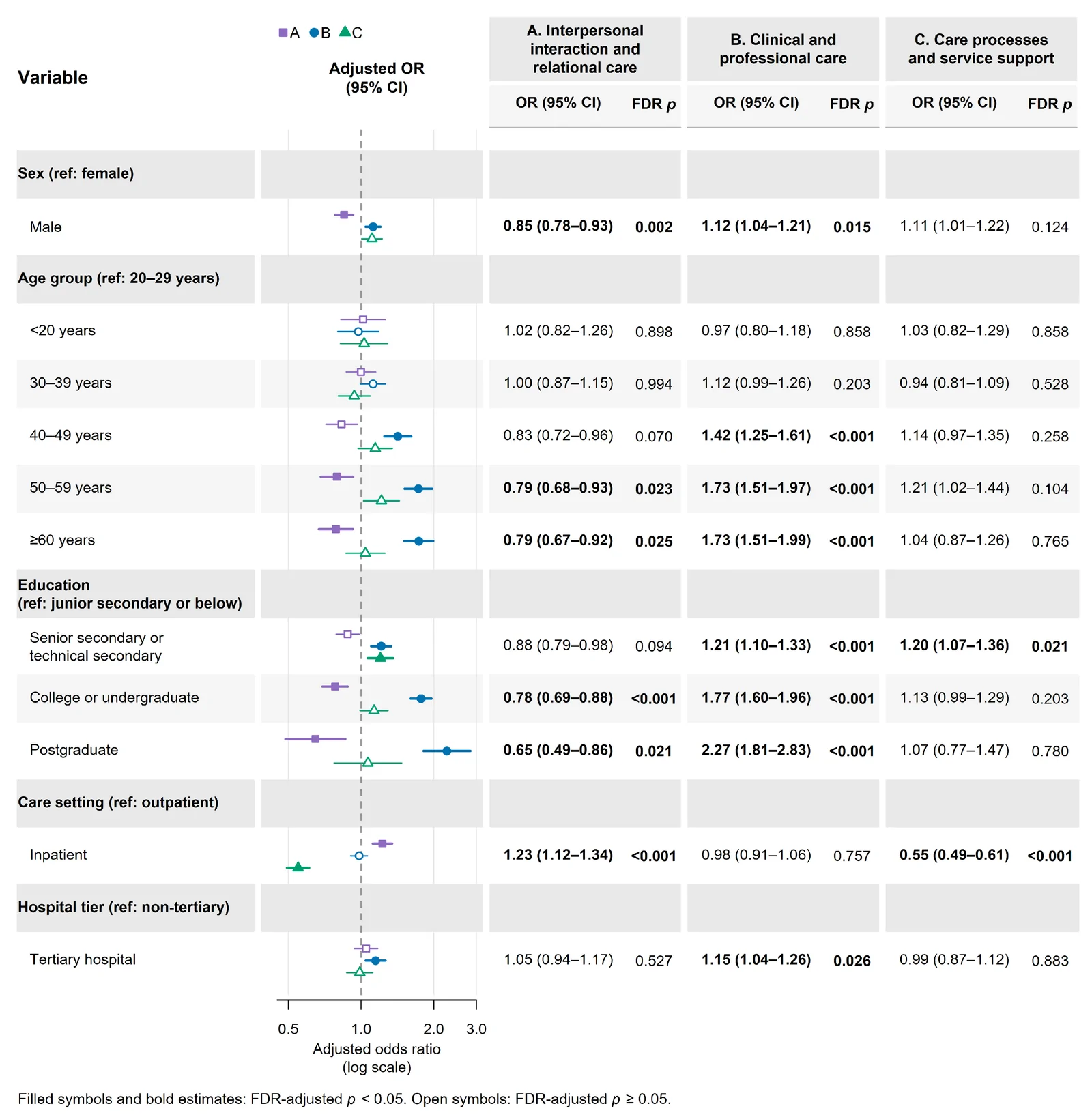
Adjusted associations of patient and care characteristics with mentions of the three care domains. Note: Purple squares (A), blue circles (B) and green triangles (C) represent interpersonal interaction and relational care, clinical and professional care, and care processes and service support, respectively. Symbols show adjusted odds ratios (ORs), and horizontal lines show conventional 95% confidence intervals (CIs) on a logarithmic scale. The dashed vertical line denotes OR = 1. Corresponding ORs, 95% CIs and FDR-adjusted *p* values are reported in the adjacent columns. Filled symbols, thicker lines and bold numerical entries indicate Benjamini–Hochberg FDR-adjusted *p* < 0.05; open symbols, thinner lines and regular-weight numerical entries indicate FDR-adjusted *p* ≥ 0.05. Adjustment covered all 150 estimable non-intercept fixed-effect coefficients across the three primary models, including coefficients not displayed here. Results are from two-level binary logistic regression models with hospital random intercepts (27,098 comments from 6580 hospitals). All three models adjusted for sex, age group, educational attainment, insurance/payment type, care setting, hospital tier, hospital type, comment length and province fixed effects. Reference groups are shown in the variable headings. Domains are not mutually exclusive. Complete results are provided in Supplementary Table S5.

All three primary models included 27,098 comments and adjusted for patient characteristics, care setting, hospital characteristics, comment length and province. Selected adjusted associations are shown in Figure 3, and complete model results are provided in Supplementary Table S5. Statistical significance in the primary and sensitivity analyses below was assessed using FDR-adjusted *p* values.

For clinical and professional care, comments from male patients had higher odds of mentioning this domain than those from female patients (adjusted OR 1.12, 95% CI 1.04–1.21). Compared with the 20–29-year group, the 40–49-year, 50–59-year and ≥60-year groups had higher odds of mentioning this domain, with adjusted ORs of 1.42 (95% CI 1.25–1.61), 1.73 (95% CI 1.51–1.97) and 1.73 (95% CI 1.51–1.99), respectively. Compared with the junior secondary or below group, those with senior secondary or technical secondary education, college or undergraduate education, and postgraduate education had higher odds of mentioning the domain, with adjusted ORs of 1.21 (95% CI 1.10–1.33), 1.77 (95% CI 1.60–1.96) and 2.27 (95% CI 1.81–2.83), respectively. Comments from tertiary hospitals had higher odds of mentioning this domain than those from non-tertiary hospitals (adjusted OR 1.15, 95% CI 1.04–1.26). The difference between inpatient and outpatient comments was not statistically significant (adjusted OR 0.98, 95% CI 0.91–1.06).

For interpersonal interaction and relational care, comments from male patients had lower odds of mentioning this domain than those from female patients (adjusted OR 0.85, 95% CI 0.78–0.93). Compared with the 20–29-year group, the 50–59-year and ≥60-year groups had lower odds, with adjusted ORs of 0.79 (95% CI 0.68–0.93) and 0.79 (95% CI 0.67–0.92), respectively. Compared with the junior secondary or below group, those with college or undergraduate education and postgraduate education had lower odds, with adjusted ORs of 0.78 (95% CI 0.69–0.88) and 0.65 (95% CI 0.49–0.86), respectively. Inpatient comments had higher odds of mentioning this domain than outpatient comments (adjusted OR 1.23, 95% CI 1.12–1.34). The associations for the 40–49-year group and the senior secondary or technical secondary education group did not reach FDR-adjusted statistical significance, with adjusted *p* values of 0.070 and 0.094, respectively. The association with hospital tier was also not statistically significant (adjusted OR 1.05, 95% CI 0.94–1.17).

For care processes and service support, inpatient comments had lower odds of mentioning this domain than outpatient comments (adjusted OR 0.55, 95% CI 0.49–0.61). Compared with the junior secondary or below group, the senior secondary or technical secondary education group had higher odds (adjusted OR 1.20, 95% CI 1.07–1.36). The associations for male patients and the 50–59-year group did not reach FDR-adjusted statistical significance, with adjusted *p* values of 0.124 and 0.104, respectively. The association with hospital tier was also not statistically significant (adjusted OR 0.99, 95% CI 0.87–1.12).

### 3.6. Sensitivity Analyses

Restricting the sample to secondary and tertiary hospitals yielded 27,088 comments from 6571 hospitals. The directions and FDR-adjusted statistical significance of the main associations in each domain were consistent with those in the full-sample random-intercept models.

In single-level logistic regression with hospital-clustered robust standard errors, inpatient comments had higher odds of mentioning interpersonal interaction and relational care than outpatient comments (adjusted OR 1.23, 95% CI 1.07–1.41; FDR-adjusted *p* = 0.041) and lower odds of mentioning care processes and service support (adjusted OR 0.53, 95% CI 0.47–0.61; FDR-adjusted *p* < 0.001). Compared with their respective reference groups, all age groups of 40 years or older and the senior secondary or technical secondary, college or undergraduate, and postgraduate education groups remained positively associated with mentions of clinical and professional care, with statistical significance retained.

The statistical significance of some associations varied by model specification: the association between tertiary hospital tier and mentions of clinical and professional care, and some associations with sex, age and educational attainment, did not reach FDR-adjusted statistical significance in the single-level models. Complete sensitivity analysis results are provided in Supplementary Table S5.

## 4. Discussion

### 4.1. Principal Findings

This nationwide study identified patient-recognised care practices in open-ended patient satisfaction survey comments using a framework of three domains and 13 categories. Among 115,466 screened comments, 75.35% expressed general praise and 27,698 described specific positive experiences assigned to care-practice categories. The analysis produced three main findings. First, the interpersonal interaction and relational care domain was mentioned most frequently, with service attitude and responsibility being particularly prominent. Second, inpatient comments had higher adjusted odds of mentioning interpersonal interaction and relational care and lower odds of mentioning care processes and service support than outpatient comments. Third, all age groups of 40 years or older more often mentioned clinical and professional care than the 20–29-year group, as did each higher educational attainment group compared with the junior secondary or below group. These associations remained statistically significant after FDR adjustment in both the primary and sensitivity analyses.

### 4.2. Interpretation

Interpersonal interaction and relational care formed a prominent category of specific positive experiences, consistent with previous studies of patient compliments. Communication, respect and caring are directly experienced by patients and shape their evaluations of healthcare alongside technical quality [22,23,24]. Patients can directly experience whether staff listen patiently, explain clearly and respond to their needs, whereas judging the appropriateness of diagnosis or treatment generally requires more professional knowledge. This difference may explain why service attitude, responsibility, communication or explanation, and emotional care appeared frequently in positive comments. Previous analyses of compliment letters found that patients often praised the caring actions and proactive assistance of individual staff members [25], and research on nursing and midwifery compliments also described care behaviours recognised by patients [26]. A study of positive patient and family feedback about the diagnostic process further identified clear communication, listening and emotional support among the commonly mentioned aspects [36]. Similar findings in our routine survey comments suggest that patients’ praise often concerns how staff treat them, explain problems and provide help during everyday encounters.

By comparison, care processes and service support were mentioned less frequently. This may reflect the foundational role of these services in the patient journey. Appointment systems, navigation, payment, the care environment and coordination between service stages are necessary for patients to complete care smoothly. When these services function well, patients may regard them as expected parts of care. When waits are long, directions are unclear or service stages are poorly coordinated, patients may be more likely to include these experiences in feedback about problems. Previous complaint studies frequently identified waiting and service organisation as concerns [12,37,38,39]. Differences in how patients describe smooth care and difficulties may partly explain why care processes and service support appear less often in positive comments.

Differences in care processes and staff–patient contact between outpatient and inpatient settings may influence the experiences patients emphasise in comments. Outpatient encounters are brief, and patients often need to complete consultations, examinations, payment and medication collection within a limited period. Clear guidance and smooth transitions between service stages may therefore be more readily described in outpatient comments. Inpatient stays involve longer and more frequent contact with healthcare staff, including repeated ward rounds, treatments, nursing care and examinations. These repeated encounters provide more opportunities to experience staff responses to discomfort, explanations of treatment, and caring and assistance in everyday care. A qualitative study of hospital stays also identified communication, timely responses and attention to individual needs as important themes [40]. Differences in these forms of contact offer possible explanations for the greater mention of relational care in inpatient comments and of care processes and service support in outpatient comments.

Positive comments from different hospital tiers showed both differences and common features. In descriptive comparisons, professional competence was mentioned more frequently in comments from tertiary hospitals. The broader clinical and professional care domain was also positively associated with tertiary hospital tier in the primary models, although this association was not statistically significant after FDR adjustment in the models with hospital-clustered robust standard errors. Differences in patient case mix and hospital service functions are possible explanations for further investigation. By contrast, service attitude was mentioned at similar rates in tertiary and non-tertiary hospital comments, at approximately 65% in each. A study of Chinese patient compliments and complaints also identified friendliness, respect, patience and responsibility [41]. At the domain level, adjusted ORs for hospital tier were close to 1 for interpersonal interaction and relational care and for care processes and service support, with neither association reaching FDR-adjusted statistical significance in the primary or sensitivity analyses. These consistent results did not indicate clear hospital-tier differences in mentions of these two domains.

At the patient level, the adjusted odds of mentioning clinical and professional care were higher in the groups aged 40 years or older than in the 20–29-year group and in each higher educational attainment group than in the junior secondary or below group. These differences may relate to previous healthcare use, exposure to health information and expectations of care, which could influence the experiences patients choose to describe. A previous survey found that the extent to which patients’ expectations were met was associated with satisfaction [42]. Whether expectations, health literacy and previous healthcare use help explain the age and education differences observed here warrants investigation using direct measures of these factors.

### 4.3. Implications for Research and Practice

Distinguishing general praise from specific care practices helps identify what patients explicitly commend. General praise expresses approval but usually does not describe what happened; comments about specific practices provide details of how staff explain problems, respond to needs and offer assistance. Previous research has highlighted the importance of content and context when interpreting and using positive feedback [27]. By organising these practices into three domains and 13 categories while retaining multiple practices within a comment, this study allows dispersed positive feedback to be classified and compared using consistent criteria.

Previous research on nursing and midwifery compliments found variation across hospital sites and service units and called for further examination of patient and hospital factors [26]. By linking classified practices with patient and hospital information, this study examines differences in mentions across patient groups, care settings and hospital tiers in national survey data. The differences between inpatient and outpatient comments, and the associations of age and educational attainment with clinical and professional care mentions, support interpreting feedback in relation to patient composition and care context. Retaining these group-specific results alongside overall summaries can help healthcare organisations identify the aspects of care frequently praised by different patient groups and in different care settings.

Healthcare organisations can share categorised positive comments and relevant excerpts with clinical teams to discuss communication, assistance and service arrangements recognised by patients and exchange practices relevant to their own services. Alongside patient experience questionnaires and care-process indicators, these accounts can inform service improvement discussions, exchange of practical experience and staff training [15,43,44,45,46,47]. Feedback collection could also invite patients to explain what happened and which actions helped them, while retaining opportunities for free expression, to increase the detail available for analysis and learning [27]. Whether the care practices identified in positive feedback lead to better patient experience or clinical or organisational outcomes warrants evaluation in prospective intervention studies.

### 4.4. Strengths, Limitations and Future Research

This study has several strengths. First, it used data from a national patient satisfaction survey covering 31 provincial-level regions of China and included different patient groups, care settings and hospital tiers, providing a common data source for comparing patient-recognised practices across care contexts. Second, the coding scheme underwent two rounds of human coding for refinement and independent validation, with a weighted micro-averaged F1 score of 95.82% (95% CI 92.63–97.66%) for 13-category coding in the second round. Third, multi-label coding retained multiple care practices mentioned within the same comment, allowing comment content to be classified and compared using consistent definitions. Fourth, multilevel regression models accounted for the clustering of comments within hospitals and adjusted for patient characteristics, care setting, hospital characteristics and province, extending the description of feedback content to an analysis of its associations with patient and care characteristics.

Several limitations should be considered. First, voluntary commenting, model screening and care-practice classification shaped the analytical sample, limiting its representativeness of positive patient feedback overall. Mention frequencies reflect the content spontaneously expressed by patients and should not be equated with perceived importance, overall satisfaction or objective quality of care [48]. Second, although the screening model and care-practice coding were each evaluated (Supplementary Tables S3 and S6), validation of the 13-category classification was limited to comments retained by model-assisted screening. These evaluations therefore do not establish the performance of the complete pipeline from the original survey comments to the final care-practice categories. False-negative classifications by the initial screening model may have excluded some relevant positive comments from subsequent coding and its validation sample. If the likelihood of such omissions differs across patient groups or care-practice categories, mention rates and association estimates could be affected. Infrequent categories, including appointment scheduling, one-stop convenience and payment and settlement, require cautious interpretation, particularly where positive validation examples were few. Finally, the cross-sectional design precludes causal inference, and unmeasured disease severity, length of stay, previous healthcare use, health literacy and patient expectations may contribute to residual confounding. All data came from China; differences in culture, service organisation and feedback practices may limit generalisability to other healthcare systems.

Future multicentre studies could link patient comments with clinical information and expectations of care to further explain feedback differences across patient groups and care settings. Building on this work, cluster-randomised trials with outpatient clinics or wards as the units of randomisation could compare usual feedback, categorised feedback, and categorised feedback combined with team discussion, assessing changes in patient experience and care-process indicators. Recording how teams interpret feedback, choose improvement measures and implement them in daily work could help examine how organisational support and care setting influence implementation effects. Continued follow-up could assess whether improvements are sustained, identifying effective ways to use categorised feedback and the conditions under which they are applicable [19].

## 5. Conclusions

This cross-sectional text analysis used 215,690 patient survey comments from 31 provincial-level regions of China and identified 27,698 care-practice comments among 115,466 comments classified as positive through model-assisted screening. General praise was the most common class among screened positive comments, followed by specific positive experiences assigned to care-practice categories. Interpersonal interaction and relational care formed the most frequently mentioned domain in care-practice comments, particularly service attitude and responsibility. Patient age, educational attainment and outpatient or inpatient setting were associated with the care content mentioned, suggesting that positive feedback should be analysed in relation to patient composition and care context. Classifying specific practices recognised by patients provides a basis for clinical teams to compare feedback content and exchange care experiences. Future prospective intervention studies could assess whether and under what conditions the identified care practices, as well as the use of categorised feedback and team discussion, improve patient experience and care processes.

## Figures and Tables

**Table 1:** Characteristics of the comment samples.

Characteristic	Category	Full Sample *n* = 215,690 *n* (%)	Care-Practice Comments *n* = 27,698 *n* (%)	Regression Sample *n* = 27,098 *n* (%)
**Sex**	Female	122,937 (57.0)	16,250 (58.7)	15,907 (58.7)
	Male	92,753 (43.0)	11,448 (41.3)	11,191 (41.3)
**Age group**	<20 years	17,562 (8.1)	1680 (6.1)	1651 (6.1)
	20–29 years	45,610 (21.1)	4418 (16.0)	4323 (16.0)
	30–39 years	66,849 (31.0)	7050 (25.5)	6884 (25.4)
	40–49 years	35,491 (16.5)	5193 (18.7)	5055 (18.7)
	50–59 years	26,111 (12.1)	4879 (17.6)	4771 (17.6)
	≥60 years	24,067 (11.2)	4478 (16.2)	4414 (16.3)
**Educational attainment**	Junior secondary or below	71,197 (33.0)	10,709 (38.7)	10,491 (38.7)
	Senior secondary or technical secondary	59,440 (27.6)	7753 (28.0)	7566 (27.9)
	College or undergraduate	77,066 (35.7)	8597 (31.0)	8418 (31.1)
	Postgraduate	7987 (3.7)	639 (2.3)	623 (2.3)
**Insurance/payment type**	Self-pay	62,132 (28.8)	5772 (20.8)	5610 (20.7)
	Publicly funded medical care	9254 (4.3)	1105 (4.0)	1080 (4.0)
	Urban employee/resident basic medical insurance	83,393 (38.7)	12,154 (43.9)	11,918 (44.0)
	New Rural Cooperative Medical Scheme	60,911 (28.2)	8667 (31.3)	8490 (31.3)
**Care setting**	Outpatient	100,887 (46.8)	11,618 (41.9)	11,336 (41.8)
	Inpatient	114,803 (53.2)	16,080 (58.1)	15,762 (58.2)
**Hospital type**	General hospital	115,546 (58.8)	16,678 (61.2)	16,655 (61.5)
	Traditional Chinese medicine hospital	30,061 (15.3)	4384 (16.1)	4350 (16.1)
	Maternal and child health institution	27,085 (13.8)	3220 (11.8)	3135 (11.6)
	Specialised or other hospital	16,966 (8.6)	2114 (7.8)	2101 (7.8)
	Integrated Chinese and Western medicine hospital	3945 (2.0)	440 (1.6)	439 (1.6)
	Ethnic medicine hospital	2861 (1.5)	429 (1.6)	418 (1.5)
**Hospital tier**	Non-tertiary	85,480 (43.5)	13,026 (47.8)	12,938 (47.7)
	Tertiary	111,037 (56.5)	14,201 (52.2)	14,160 (52.3)

Note: Values are numbers of comments and percentages. Percentages use the number of comments with available data for each characteristic as the denominator. Hospital type was available for 196,464 comments in the full sample, 27,265 in the care-practice comments and all 27,098 in the regression sample; hospital tier was available for 196,517, 27,227 and 27,098 comments, respectively. Full-sample hospital characteristics use the recorded survey categories; hospital characteristics for care-practice comments and the regression sample use the reviewed hospital classifications. In the full sample, non-tertiary hospitals also include the recorded category “primary and other”. Percentages may not sum to 100% because of rounding. Bold text in the first column indicates characteristic groups.

**Table 2:** Mentions of care-practice domains and categories among care-practice comments and all screened comments.

Domain/Category	*n*	Care-Practice Comments (%) *n* = 27,698	Screened Comments (%) *n* = 115,466
**Interpersonal interaction and relational care**	**24,883**	**89.84**	**21.55**
Service attitude	17,984	64.93	15.58
Responsibility	6507	23.49	5.64
Communication or explanation	3612	13.04	3.13
Emotional care	2556	9.23	2.21
Proactive help	739	2.67	0.64
**Clinical and professional care**	**4** **684**	**16.91**	**4.06**
Professional competence	4024	14.53	3.49
Timely effort	761	2.75	0.66
**Care processes and service support**	**2** **392**	**8.64**	**2.07**
Environmental cleanliness	1216	4.39	1.05
Navigation and guidance	367	1.33	0.32
Smooth process	804	2.90	0.70
One-stop convenience	29	0.10	0.03
Appointment scheduling	17	0.06	0.01
Payment and settlement	34	0.12	0.03

Note: Counts are numbers of comments mentioning each domain or category. Percentages use the 27,698 comments assigned to at least one care-practice category or all 115,466 screened comments as the denominator, as indicated in the column headings. Domain rows indicate comments mentioning at least one category within that domain; category rows indicate comments mentioning the specific category. Categories and domains are not mutually exclusive, so percentages may sum to more than 100%. Bold rows indicate domain-level results.

## Data Availability

The de-identified free-text comments and individual-level analytic data are not publicly available because of privacy and data-use restrictions. Aggregated results supporting the findings are provided in the article and Supplementary Materials. The analysis code and coding rules are available from the corresponding author upon reasonable request.

## Supplementary Materials

The following supporting information can be downloaded at https://www.mdpi.com/article/10.3390/healthcare14203422/s1, Supplementary Table S1, operational definitions and illustrative expressions for the 13 care-practice categories; Supplementary Table S2, inter-coder agreement; Supplementary Table S3, coding performance against the human reference standard; Supplementary Table S4, descriptive comparisons of category mention rates by care setting and hospital tier; Supplementary Table S5, complete primary and sensitivity analysis results; Supplementary Table S6, performance of the iFLYTEK Spark model for topic and sentiment classification.
