# Supplementary material for: Identifying Patient-Recognised Care Practices and Variation in Positive Feedback Content: A Nationwide Cross-Sectional Text Analysis in China
Source: Healthcare (Basel). 2026 Oct 13;14(20):3422. doi: 10.3390/healthcare14203422 (PMC13704691; doi:10.3390/healthcare14203422)
Supplement: Supplementary file 1 [file healthcare-14-03422-s001.zip › healthcare-4514657-supplementary.pdf]

Supplementary Materials

Identifying Patient-Recognised Care Practices and Variation in Positive Feedback Content: A Nationwide Cross-Sectional Text Analysis in China

Supplementary Table S1. Definitions and operational coding rules for the 13 patient-recognised care-practice categories

Domain	Category	Operational definition	Illustrative positive expressions
Interpersonal interaction and relational care	Service attitude	Positive mentions of warmth, friendliness, courtesy, respect or a helpful service manner.	warm; friendly; respectful; good service attitude
Interpersonal interaction and relational care	Responsibility	Positive mentions of careful, conscientious, responsible or attentive care, including appropriate follow-up.	careful; responsible; conscientious; attentive
Interpersonal interaction and relational care	Communication or explanation	Positive mentions of clear explanations, patient communication, detailed advice or responses to questions.	clear explanation; patient explanation; easy to understand
Interpersonal interaction and relational care	Emotional care	Positive mentions of reassurance, comfort, empathy, emotional support or family-like care.	reassuring; comforting; made me feel safe
Interpersonal interaction and relational care	Proactive help	Positive mentions of staff taking the initiative to provide practical assistance beyond routine instructions.	offered help; accompanied me; helped with procedures
Clinical and professional care	Professional competence	Positive mentions of clinical skill, professional judgement, diagnostic or treatment quality, or a favourable treatment or recovery outcome perceived by the patient.	skilled; professional; accurate diagnosis; treatment worked well
Clinical and professional care	Timely effort	Positive mentions of prompt response, timely treatment, rapid rescue or staff making additional effort to provide care.	prompt; timely; rapid response; made every effort
Care processes and service support	Environmental cleanliness	Positive mentions of cleanliness, comfort, a pleasant care environment or adequate facilities.	clean; comfortable; pleasant environment; good facilities
Care processes and service support	Navigation and guidance	Positive mentions of useful guidance from navigation, triage, front-desk or volunteer staff.	clear guidance; helpful triage; navigation support
Care processes and service support	Smooth process	Positive mentions of efficient, convenient, orderly or well-coordinated care processes with little waiting or repetition.	efficient; convenient care process; smooth process; little waiting
Care processes and service support	One-stop convenience	Positive mentions of integrated or one-stop services that reduced repeated visits between departments or service points.	one-stop service; several services completed together
Care processes and service support	Appointment scheduling	Positive mentions of convenient, successful or well-organised appointment scheduling, including time-slot appointments.	easy appointment; time-slot booking; smooth scheduling
Care processes and service support	Payment and settlement	Positive mentions of convenient, rapid or transparent payment, charging or settlement processes.	easy payment; rapid settlement; clear charges

Note: Coding was multi-label; one comment could be assigned to more than one category. Examples illustrate meanings and are not verbatim quotations or an exhaustive list of rules. Coding required an explicitly described positive practice; requests and wishes were not treated as practices received. For comments also containing criticism or suggestions, only positive practices supported by the original text were labelled. Topic classifications were not used to infer unexpressed practices. A domain was present when at least one of its component categories was present.

Supplementary Table S2. Inter-coder agreement in the independent human validation sample (n = 600)

Classification	Unweighted agreement, %	Unweighted Cohen's kappa	Weighted agreement, %	Weighted Cohen's kappa	Disagreements, n
Five main classes	91.33	0.880	97.21	0.927	52
Service attitude	98.33	0.944	97.89	0.916	10
Responsibility	99.83	0.990	99.83	0.984	1
Communication or explanation	99.83	0.986	99.83	0.975	1
Emotional care	99.83	0.982	100.00	1.000	1
Proactive help	99.83	0.969	99.88	0.912	1
Professional competence	99.67	0.970	100.00	1.000	2
Timely effort	99.33	0.885	99.52	0.715	4
Environmental cleanliness	99.67	0.927	99.92	0.959	2
Navigation and guidance	99.67	0.898	99.97	0.940	2
Smooth process	98.00	0.831	99.73	0.769	12
One-stop convenience	99.17	0.894	100.00	0.898	5
Appointment scheduling	99.67	0.939	100.00	0.986	2
Payment and settlement	99.83	0.984	100.00	0.985	1

Note: Agreement was calculated from the two coders' independent classifications before discussion; coders did not have access to program classifications. Five-class agreement treats the main class as a nominal outcome; each care-practice category is binary. Weighted estimates use the population-to-sample ratio within each sampling stratum. Disagreement counts are unweighted. Rounded weighted agreement and kappa may equal 100.00% and 1.000 despite observed disagreements. The 600 comments comprised 150 general-praise comments, 250 practice comments, 80 positive descriptions not assigned to care-practice categories, 65 comments containing factual statements, questions or suggestions and 55 with insufficient information; practice comments were further stratified for rare labels.

Supplementary Table S3. Coding performance against the human reference standard in the independent validation sample (n = 600)

Panel A. Five main classes

Class or category	Human positive, n	Program positive, n	Weighted PPV, % (95% CI)	Weighted sensitivity, % (95% CI)	Weighted F1, % (95% CI)
A: General praise	158	150	99.33 (95.40–99.91)	99.27 (97.41–99.79)	99.30 (97.80–99.78)
B: Specific positive experiences assigned to care-practice categories	243	250	97.09 (91.95–98.98)	97.84 (87.01–99.67)	97.46 (93.22–99.07)
C: Positive descriptions not assigned to care-practice categories	86	80	96.25 (90.03–98.65)	76.43 (49.33–91.53)	85.20 (66.20–94.42)
D: Factual statements, questions and suggestions	63	65	95.38 (88.10–98.30)	82.89 (40.97–97.13)	88.70 (61.53–97.47)
E: Insufficient information	50	55	89.09 (NE)	90.28 (61.41–98.19)	89.68 (79.20–95.20)

Panel B. Overall classification performance

Measure	Unweighted estimate, %	Weighted estimate, % (95% CI)
Five-class accuracy	96.00	98.77 (96.70–99.55)
13-label micro-averaged PPV	91.76	95.41 (91.71–97.50)
13-label micro-averaged sensitivity	96.80	96.23 (90.80–98.51)
13-label micro-averaged F1	94.21	95.82 (92.63–97.66)
13-label macro-averaged F1	93.64	93.87 (90.33–96.17)

Panel C. Thirteen care-practice categories

Class or category	Human positive, n	Program positive, n	Weighted PPV, % (95% CI)	Weighted sensitivity, % (95% CI)	Weighted F1, % (95% CI)
Service attitude	109	122	92.97 (85.28–96.80)	96.72 (80.57–99.52)	94.81 (88.70–97.70)
Responsibility	53	55	96.29 (83.34–99.26)	100.00 (NE)	98.11 (90.91–99.63)
Communication or explanation	40	38	100.00 (NE)	95.25 (74.02–99.30)	97.57 (85.07–99.65)
Emotional care	30	29	99.96 (99.96–99.97)	97.96 (87.47–99.70)	98.95 (93.41–99.84)
Proactive help	17	16	100.00 (NE)	83.87 (42.40–97.35)	91.23 (59.55–98.66)
Professional competence	35	34	99.98 (99.97–99.98)	93.00 (65.77–98.92)	96.36 (79.40–99.45)
Timely effort	18	19	91.61 (57.60–98.87)	75.97 (30.75–95.75)	83.06 (48.95–96.16)
Environmental cleanliness	14	15	99.92 (99.91–99.92)	100.00 (NE)	99.96 (99.95–99.96)
Navigation and guidance	10	12	72.80 (40.50–91.32)	99.63 (99.46–99.74)	84.13 (57.65–95.38)
Smooth process	38	41	86.09 (58.62–96.43)	98.34 (95.24–99.43)	91.80 (74.19–97.76)
One-stop convenience	24	29	82.76 (NE)	100.00 (NE)	90.57 (NE)
Appointment scheduling	15	17	88.24 (NE)	100.00 (NE)	93.75 (NE)
Payment and settlement	34	34	100.00 (NE)	100.00 (NE)	100.00 (NE)

Note: Positive counts are unweighted and refer to the validation sample, not to the full corpus. The human reference standard was established after discussion of disagreements. Performance estimates use sampling-stratum weights. PPV, positive predictive value; CI, confidence interval; NE, confidence interval not reliably estimable because the estimate was at a boundary or its estimated sampling variance was zero. Available 95% CIs use Taylor linearisation with stratified finite-population correction and a logit transformation. Low positive counts limit the interpretation of category-specific performance, even when point estimates are high.

Supplementary Table S4. Descriptive mention rates of the 13 care-practice categories by care setting and hospital tier

Category	Outpatient n = 11,336	Inpatient n = 15,762	Non-tertiary hospital n = 12,938	Tertiary hospital n = 14,160
Service attitude	7,520 (66.34)	10,062 (63.84)	8,411 (65.01)	9,171 (64.77)
Responsibility	2,431 (21.44)	3,936 (24.97)	2,648 (20.47)	3,719 (26.26)
Communication or explanation	1,634 (14.41)	1,913 (12.14)	1,616 (12.49)	1,931 (13.64)
Emotional care	527 (4.65)	1,978 (12.55)	857 (6.62)	1,648 (11.64)
Proactive help	322 (2.84)	402 (2.55)	318 (2.46)	406 (2.87)
Professional competence	1,538 (13.57)	2,394 (15.19)	1,681 (12.99)	2,251 (15.90)
Timely effort	335 (2.96)	411 (2.61)	337 (2.60)	409 (2.89)
Environmental cleanliness	390 (3.44)	799 (5.07)	553 (4.27)	636 (4.49)
Navigation and guidance	323 (2.85)	27 (0.17)	129 (1.00)	221 (1.56)
Smooth process	548 (4.83)	233 (1.48)	403 (3.11)	378 (2.67)
One-stop convenience	22 (0.19)	7 (0.04)	13 (0.10)	16 (0.11)
Appointment scheduling	14 (0.12)	3 (0.02)	9 (0.07)	8 (0.06)
Payment and settlement	18 (0.16)	15 (0.10)	22 (0.17)	11 (0.08)

Note: Values are n (%); each percentage uses its corresponding group denominator. Both comparisons use the same regression sample of 27,098 comments. Categories are not mutually exclusive. These are unadjusted descriptive proportions; no category-level hypothesis tests were performed in the current analysis. Adjusted associations for the three domains are reported in Table S5.

Supplementary Table S5. Complete primary and sensitivity regression results

Three outcomes were analysed: interpersonal interaction and relational care (I), clinical and professional care (C), and care processes and service support (P). Each outcome indicated the mention of at least one category within the corresponding domain. Panels A–D report all fixed-effect coefficients, including province indicators and intercepts.

Note: OR, odds ratio; CI, confidence interval; q, Benjamini–Hochberg FDR-adjusted P value. Within each panel, adjustment jointly covered all 150 estimable non-intercept fixed-effect coefficients across the three outcomes. Intercepts were excluded from FDR adjustment. Conventional 95% CIs describe the precision of individual association estimates; statistical significance is assessed using FDR-adjusted P values. Each model adjusted for all characteristics shown. Reference categories are indicated in the table; province comparisons use Shanghai.

Panel A. Primary hospital random-intercept models

Sample: 27,098 comments from 6,580 hospitals. Outcome-positive comments: I, 24,343; C, 4,578; P, 2,324. All three models converged.

Characteristic	I<br>OR (95% CI)	I<br>P	I<br>q	C<br>OR (95% CI)	C<br>P	C<br>q	P<br>OR (95% CI)	P<br>P	P<br>q
Sex: female (reference)	Reference	—	—	Reference	—	—	Reference	—	—
Sex: male	0.85 (0.78–0.93)	<0.001	0.002	1.12 (1.04–1.21)	0.002	0.015	1.11 (1.01–1.22)	0.034	0.124
Age: <20 years	1.02 (0.82–1.26)	0.862	0.898	0.97 (0.80–1.18)	0.794	0.858	1.03 (0.82–1.29)	0.800	0.858
Age: 20–29 years (reference)	Reference	—	—	Reference	—	—	Reference	—	—
Age: 30–39 years	1.00 (0.87–1.15)	0.989	0.994	1.12 (0.99–1.26)	0.064	0.203	0.94 (0.81–1.09)	0.401	0.528
Age: 40–49 years	0.83 (0.72–0.96)	0.014	0.070	1.42 (1.25–1.61)	<0.001	<0.001	1.14 (0.97–1.35)	0.105	0.258
Age: 50–59 years	0.79 (0.68–0.93)	0.003	0.023	1.73 (1.51–1.97)	<0.001	<0.001	1.21 (1.02–1.44)	0.026	0.104
Age: ≥60 years	0.79 (0.67–0.92)	0.004	0.025	1.73 (1.51–1.99)	<0.001	<0.001	1.04 (0.87–1.26)	0.663	0.765
Education: junior secondary or below (reference)	Reference	—	—	Reference	—	—	Reference	—	—
Education: senior or technical secondary	0.88 (0.79–0.98)	0.022	0.094	1.21 (1.10–1.33)	<0.001	<0.001	1.20 (1.07–1.36)	0.003	0.021
Education: college or undergraduate	0.78 (0.69–0.88)	<0.001	<0.001	1.77 (1.60–1.96)	<0.001	<0.001	1.13 (0.99–1.29)	0.067	0.203
Education: postgraduate	0.65 (0.49–0.86)	0.003	0.021	2.27 (1.81–2.83)	<0.001	<0.001	1.07 (0.77–1.47)	0.691	0.780
Payment: self-pay (reference)	Reference	—	—	Reference	—	—	Reference	—	—
Payment: publicly funded medical care	0.97 (0.77–1.23)	0.818	0.870	1.06 (0.87–1.28)	0.581	0.703	0.97 (0.75–1.25)	0.799	0.858
Payment: urban employee/resident basic insurance	0.96 (0.85–1.09)	0.515	0.654	1.15 (1.03–1.28)	0.011	0.061	0.98 (0.86–1.12)	0.799	0.858
Payment: New Rural Cooperative Medical Scheme	1.02 (0.90–1.16)	0.756	0.833	1.00 (0.89–1.12)	0.994	0.994	1.01 (0.88–1.17)	0.842	0.883
Care setting: outpatient (reference)	Reference	—	—	Reference	—	—	Reference	—	—
Care setting: inpatient	1.23 (1.12–1.34)	<0.001	<0.001	0.98 (0.91–1.06)	0.647	0.757	0.55 (0.49–0.61)	<0.001	<0.001
Hospital tier: non-tertiary (reference)	Reference	—	—	Reference	—	—	Reference	—	—
Hospital tier: tertiary	1.05 (0.94–1.17)	0.395	0.527	1.15 (1.04–1.26)	0.004	0.026	0.99 (0.87–1.12)	0.839	0.883

Characteristic	I<br>OR (95% CI)	I<br>P	I<br>q	C<br>OR (95% CI)	C<br>P	C<br>q	P<br>OR (95% CI)	P<br>P	P<br>q
Hospital type: general (reference)	Reference	—	—	Reference	—	—	Reference	—	—
Hospital type: traditional Chinese medicine	0.84 (0.73–0.96)	0.012	0.064	1.19 (1.06–1.34)	0.004	0.026	0.95 (0.81–1.11)	0.503	0.645
Hospital type: maternal and child health	1.01 (0.85–1.20)	0.892	0.922	0.82 (0.70–0.96)	0.014	0.070	1.09 (0.91–1.31)	0.349	0.481
Hospital type: specialised or other	0.92 (0.76–1.11)	0.381	0.515	1.21 (1.03–1.40)	0.017	0.077	0.88 (0.70–1.09)	0.232	0.386
Hospital type: integrated Chinese and Western medicine	0.62 (0.45–0.86)	0.005	0.027	1.35 (1.01–1.80)	0.042	0.147	1.29 (0.88–1.90)	0.188	0.361
Hospital type: ethnic medicine	0.48 (0.33–0.69)	<0.001	<0.001	1.82 (1.29–2.56)	<0.001	0.006	1.02 (0.63–1.66)	0.928	0.953
Comment length: per Chinese character	1.006 (1.004–1.008)	<0.001	<0.001	1.018 (1.016–1.019)	<0.001	<0.001	1.010 (1.008–1.011)	<0.001	<0.001
Province: Shanghai (reference)	Reference	—	—	Reference	—	—	Reference	—	—
Province: Yunnan	1.57 (0.96–2.57)	0.072	0.203	0.59 (0.39–0.89)	0.013	0.065	0.56 (0.32–0.97)	0.039	0.138
Province: Inner Mongolia	1.41 (0.84–2.38)	0.193	0.365	0.69 (0.45–1.08)	0.104	0.258	0.62 (0.35–1.13)	0.118	0.267
Province: Beijing	1.14 (0.66–1.95)	0.638	0.753	1.00 (0.64–1.55)	0.988	0.994	0.60 (0.32–1.11)	0.102	0.258
Province: Jilin	1.43 (0.77–2.66)	0.263	0.415	0.72 (0.43–1.20)	0.210	0.371	0.82 (0.42–1.61)	0.574	0.703
Province: Sichuan	1.48 (0.91–2.40)	0.118	0.267	0.70 (0.46–1.05)	0.083	0.226	0.65 (0.38–1.12)	0.120	0.267
Province: Tianjin	1.19 (0.59–2.38)	0.622	0.741	1.11 (0.64–1.94)	0.710	0.795	0.60 (0.26–1.37)	0.224	0.378
Province: Ningxia	1.47 (0.72–2.98)	0.287	0.439	0.72 (0.39–1.31)	0.279	0.431	0.67 (0.31–1.47)	0.321	0.467
Province: Anhui	1.40 (0.81–2.42)	0.234	0.386	0.59 (0.37–0.94)	0.027	0.104	0.88 (0.49–1.61)	0.688	0.780
Province: Shandong	1.58 (0.96–2.62)	0.072	0.203	0.76 (0.51–1.16)	0.204	0.371	0.76 (0.44–1.31)	0.326	0.471
Province: Shanxi	1.68 (0.96–2.93)	0.069	0.203	0.50 (0.31–0.81)	0.005	0.027	0.65 (0.35–1.19)	0.163	0.320
Province: Guangdong	1.42 (0.88–2.28)	0.150	0.308	0.73 (0.49–1.09)	0.121	0.267	0.72 (0.42–1.21)	0.215	0.375
Province: Guangxi	1.38 (0.83–2.29)	0.209	0.371	0.66 (0.43–1.01)	0.057	0.186	0.81 (0.46–1.41)	0.457	0.597
Province: Xinjiang	1.29 (0.77–2.15)	0.339	0.481	0.83 (0.54–1.28)	0.397	0.527	0.72 (0.41–1.28)	0.266	0.415
Province: Jiangsu	1.64 (0.96–2.79)	0.071	0.203	0.76 (0.49–1.18)	0.223	0.378	0.66 (0.36–1.18)	0.163	0.320
Province: Jiangxi	1.37 (0.81–2.31)	0.245	0.398	0.89 (0.57–1.38)	0.607	0.728	0.88 (0.49–1.56)	0.651	0.757
Province: Hebei	1.55 (0.94–2.58)	0.088	0.229	0.79 (0.52–1.20)	0.264	0.415	0.66 (0.38–1.15)	0.142	0.300
Province: Henan	1.39 (0.84–2.29)	0.195	0.365	0.69 (0.45–1.05)	0.083	0.226	0.78 (0.45–1.36)	0.380	0.515
Province: Zhejiang	1.17 (0.70–1.95)	0.554	0.699	0.85 (0.56–1.31)	0.473	0.612	0.76 (0.43–1.35)	0.349	0.481
Province: Hainan	1.96 (0.96–4.00)	0.065	0.203	0.31 (0.16–0.61)	<0.001	0.006	0.81 (0.38–1.71)	0.573	0.703
Province: Hubei	1.32 (0.78–2.25)	0.302	0.453	0.73 (0.46–1.14)	0.164	0.320	0.89 (0.49–1.59)	0.689	0.780
Province: Hunan	1.49 (0.88–2.53)	0.138	0.295	0.65 (0.42–1.01)	0.056	0.186	0.64 (0.36–1.15)	0.133	0.290
Province: Gansu	1.29 (0.79–2.12)	0.312	0.459	0.76 (0.50–1.16)	0.210	0.371	0.74 (0.43–1.29)	0.293	0.443
Province: Fujian	1.42 (0.81–2.51)	0.221	0.378	0.66 (0.41–1.06)	0.088	0.229	0.84 (0.45–1.56)	0.577	0.703

Characteristic	I OR (95% CI)	I P	I q	C OR (95% CI)	C P	C q	P OR (95% CI)	P P	P q
Province: Tibet	0.98 (0.50–1.95)	0.964	0.984	0.60 (0.31–1.13)	0.115	0.267	0.54 (0.22–1.28)	0.160	0.320
Province: Guizhou	1.54 (0.91–2.59)	0.108	0.258	0.58 (0.37–0.91)	0.017	0.077	0.85 (0.48–1.50)	0.576	0.703
Province: Liaoning	1.61 (0.93–2.78)	0.088	0.229	0.60 (0.38–0.94)	0.027	0.104	0.90 (0.50–1.62)	0.719	0.799
Province: Chongqing	1.40 (0.79–2.48)	0.247	0.398	0.80 (0.49–1.28)	0.346	0.481	0.59 (0.31–1.12)	0.108	0.258
Province: Shaanxi	1.80 (1.06–3.07)	0.030	0.113	0.80 (0.52–1.23)	0.308	0.457	0.76 (0.42–1.35)	0.342	0.481
Province: Qinghai	2.03 (1.09–3.79)	0.027	0.104	0.41 (0.24–0.71)	0.002	0.014	0.65 (0.33–1.28)	0.209	0.371
Province: Heilongjiang	1.85 (1.01–3.40)	0.047	0.161	0.70 (0.43–1.14)	0.147	0.305	0.43 (0.22–0.86)	0.017	0.077
Intercept (exponentiated)	9.24 (5.70–14.96)	<0.001	—	0.07 (0.04–0.10)	<0.001	—	0.09 (0.05–0.15)	<0.001	—

Model log likelihood: I, -8560.286; C, -11197.275; P, -7476.149.

Hospital random-intercept variance (95% CI): I, 0.429 (0.332–0.554); C, 0.425 (0.351–0.515); P, 0.863 (0.715–1.043).

Intraclass correlation coefficient (95% CI): I, 0.115 (0.092–0.144); C, 0.114 (0.096–0.135); P, 0.208 (0.178–0.241).

Panel B. Single-level logistic models with hospital-clustered standard errors

Sample: 27,098 comments from 6,580 hospitals. Outcome-positive comments: I, 24,343; C, 4,578; P, 2,324. All three models converged.

Characteristic	I OR (95% CI)	I P	I q	C OR (95% CI)	C P	C q	P OR (95% CI)	P P	P q
Sex: female (reference)	Reference	—	—	Reference	—	—	Reference	—	—
Sex: male	0.85 (0.76–0.96)	0.007	0.053	1.14 (1.06–1.23)	<0.001	0.009	1.09 (0.97–1.21)	0.144	0.299
Age: <20 years	1.05 (0.80–1.37)	0.736	0.836	0.98 (0.82–1.18)	0.850	0.928	1.01 (0.80–1.27)	0.963	0.974
Age: 20–29 years (reference)	Reference	—	—	Reference	—	—	Reference	—	—
Age: 30–39 years	1.00 (0.84–1.18)	0.966	0.974	1.13 (0.99–1.29)	0.072	0.221	0.93 (0.79–1.09)	0.364	0.492
Age: 40–49 years	0.85 (0.71–1.00)	0.056	0.208	1.40 (1.23–1.58)	<0.001	<0.001	1.12 (0.95–1.33)	0.175	0.316
Age: 50–59 years	0.81 (0.65–0.99)	0.045	0.181	1.69 (1.43–1.98)	<0.001	<0.001	1.16 (0.93–1.44)	0.178	0.319
Age: ≥60 years	0.78 (0.65–0.94)	0.009	0.062	1.70 (1.44–2.02)	<0.001	<0.001	1.01 (0.84–1.20)	0.934	0.960
Education: junior secondary or below (reference)	Reference	—	—	Reference	—	—	Reference	—	—
Education: senior or technical secondary	0.95 (0.85–1.06)	0.370	0.495	1.15 (1.04–1.26)	0.005	0.045	1.16 (1.01–1.32)	0.031	0.143
Education: college or undergraduate	0.88 (0.76–1.00)	0.059	0.208	1.61 (1.43–1.81)	<0.001	<0.001	1.08 (0.92–1.26)	0.340	0.472
Education: postgraduate	0.72 (0.54–0.97)	0.032	0.143	2.07 (1.63–2.62)	<0.001	<0.001	0.97 (0.70–1.34)	0.855	0.928
Payment: self-pay (reference)	Reference	—	—	Reference	—	—	Reference	—	—

Characteristic	I<br>OR (95% CI)	I<br>P	I<br>q	C<br>OR (95% CI)	C<br>P	C<br>q	P<br>OR (95% CI)	P<br>P	P<br>q
Payment: publicly funded medical care	0.94 (0.75–1.19)	0.627	0.755	1.11 (0.90–1.37)	0.330	0.462	1.01 (0.78–1.30)	0.934	0.960
Payment: urban employee/resident basic insurance	0.91 (0.80–1.03)	0.140	0.299	1.19 (1.06–1.34)	0.004	0.041	1.01 (0.86–1.19)	0.862	0.928
Payment: New Rural Cooperative Medical Scheme	1.04 (0.91–1.19)	0.566	0.708	0.99 (0.87–1.13)	0.929	0.960	1.01 (0.86–1.18)	0.912	0.960
Care setting: outpatient (reference)	Reference	—	—	Reference	—	—	Reference	—	—
Care setting: inpatient	1.23 (1.07–1.41)	0.004	0.041	0.98 (0.88–1.10)	0.761	0.859	0.53 (0.47–0.61)	<0.001	<0.001
Hospital tier: non-tertiary (reference)	Reference	—	—	Reference	—	—	Reference	—	—
Hospital tier: tertiary	1.10 (0.98–1.23)	0.117	0.280	1.08 (0.95–1.22)	0.258	0.397	1.00 (0.85–1.17)	0.975	0.975
Hospital type: general (reference)	Reference	—	—	Reference	—	—	Reference	—	—
Hospital type: traditional Chinese medicine	0.83 (0.71–0.96)	0.012	0.077	1.12 (0.98–1.28)	0.101	0.254	0.96 (0.81–1.13)	0.625	0.755
Hospital type: maternal and child health	1.28 (0.91–1.80)	0.157	0.311	0.68 (0.53–0.89)	0.004	0.041	1.01 (0.82–1.24)	0.905	0.960
Hospital type: specialised or other	1.00 (0.80–1.27)	0.967	0.974	1.10 (0.92–1.31)	0.295	0.426	0.90 (0.70–1.15)	0.382	0.507
Hospital type: integrated Chinese and Western medicine	0.67 (0.50–0.89)	0.006	0.051	1.24 (0.96–1.60)	0.101	0.254	1.26 (0.91–1.73)	0.162	0.315
Hospital type: ethnic medicine	0.38 (0.17–0.83)	0.015	0.085	2.32 (1.09–4.90)	0.028	0.137	0.98 (0.55–1.72)	0.933	0.960
Comment length: per Chinese character	1.006 (1.002–1.009)	<0.001	0.009	1.017 (1.015–1.019)	<0.001	<0.001	1.009 (1.007–1.010)	<0.001	<0.001
Province: Shanghai (reference)	Reference	—	—	Reference	—	—	Reference	—	—
Province: Yunnan	1.51 (0.97–2.37)	0.071	0.221	0.59 (0.39–0.88)	0.011	0.071	0.59 (0.36–0.96)	0.034	0.152
Province: Inner Mongolia	1.48 (0.89–2.46)	0.130	0.288	0.68 (0.44–1.06)	0.088	0.244	0.61 (0.36–1.02)	0.061	0.208
Province: Beijing	1.37 (0.71–2.67)	0.350	0.481	0.89 (0.56–1.41)	0.626	0.755	0.52 (0.28–0.97)	0.040	0.173
Province: Jilin	1.40 (0.80–2.45)	0.245	0.391	0.71 (0.44–1.15)	0.167	0.315	0.88 (0.45–1.73)	0.712	0.826
Province: Sichuan	1.41 (0.88–2.26)	0.150	0.300	0.70 (0.46–1.05)	0.087	0.244	0.69 (0.41–1.17)	0.167	0.315
Province: Tianjin	1.17 (0.61–2.24)	0.629	0.755	1.05 (0.62–1.78)	0.853	0.928	0.56 (0.28–1.11)	0.097	0.254
Province: Ningxia	1.47 (0.80–2.70)	0.219	0.359	0.71 (0.38–1.32)	0.278	0.413	0.65 (0.33–1.30)	0.222	0.359
Province: Anhui	1.37 (0.83–2.27)	0.222	0.359	0.59 (0.37–0.93)	0.024	0.126	0.85 (0.50–1.46)	0.558	0.703
Province: Shandong	1.55 (0.98–2.45)	0.061	0.208	0.75 (0.50–1.11)	0.149	0.300	0.78 (0.48–1.28)	0.323	0.457
Province: Shanxi	1.60 (0.94–2.73)	0.083	0.238	0.51 (0.33–0.79)	0.003	0.030	0.64 (0.36–1.12)	0.117	0.280
Province: Guangdong	1.36 (0.88–2.10)	0.173	0.316	0.75 (0.51–1.10)	0.146	0.299	0.74 (0.45–1.20)	0.223	0.359
Province: Guangxi	1.31 (0.83–2.06)	0.253	0.396	0.67 (0.44–1.01)	0.058	0.208	0.79 (0.49–1.29)	0.353	0.481
Province: Xinjiang	1.08 (0.61–1.92)	0.781	0.868	0.89 (0.55–1.44)	0.644	0.767	0.67 (0.41–1.12)	0.125	0.285
Province: Jiangsu	1.54 (0.96–2.49)	0.076	0.223	0.83 (0.53–1.29)	0.402	0.525	0.70 (0.36–1.35)	0.286	0.421
Province: Jiangxi	1.31 (0.79–2.18)	0.294	0.426	1.18 (0.51–2.73)	0.691	0.816	0.86 (0.49–1.53)	0.615	0.755
Province: Hebei	1.54 (0.97–2.44)	0.070	0.221	0.77 (0.52–1.14)	0.195	0.332	0.67 (0.39–1.13)	0.132	0.288

Characteristic	I OR (95% CI)	I P	I q	C OR (95% CI)	C P	C q	P OR (95% CI)	P P	P q
Province: Henan	1.56 (0.88–2.77)	0.132	0.288	0.62 (0.35–1.08)	0.090	0.244	0.66 (0.37–1.16)	0.146	0.299
Province: Zhejiang	0.73 (0.46–1.18)	0.202	0.340	1.20 (0.79–1.84)	0.388	0.511	0.53 (0.33–0.85)	0.009	0.061
Province: Hainan	1.88 (0.94–3.76)	0.075	0.223	0.31 (0.17–0.57)	<0.001	0.002	0.80 (0.39–1.64)	0.536	0.682
Province: Hubei	0.80 (0.44–1.46)	0.463	0.599	0.73 (0.49–1.09)	0.123	0.285	1.11 (0.62–1.99)	0.721	0.826
Province: Hunan	1.40 (0.84–2.32)	0.193	0.332	0.65 (0.42–0.99)	0.047	0.185	0.68 (0.40–1.17)	0.168	0.315
Province: Gansu	1.37 (0.86–2.17)	0.183	0.323	0.71 (0.47–1.07)	0.102	0.254	0.71 (0.42–1.18)	0.185	0.323
Province: Fujian	1.09 (0.60–2.00)	0.768	0.860	1.08 (0.46–2.54)	0.866	0.928	0.72 (0.40–1.28)	0.265	0.401
Province: Tibet	1.26 (0.64–2.47)	0.503	0.644	0.52 (0.26–1.04)	0.065	0.218	0.50 (0.21–1.21)	0.124	0.285
Province: Guizhou	1.51 (0.93–2.45)	0.094	0.252	0.58 (0.37–0.90)	0.014	0.085	1.14 (0.56–2.32)	0.718	0.826
Province: Liaoning	1.65 (0.99–2.76)	0.056	0.208	0.58 (0.37–0.92)	0.022	0.117	1.19 (0.49–2.86)	0.697	0.817
Province: Chongqing	1.37 (0.80–2.34)	0.253	0.396	0.78 (0.50–1.22)	0.276	0.413	0.58 (0.33–1.05)	0.070	0.221
Province: Shaanxi	1.73 (1.06–2.82)	0.028	0.137	0.81 (0.54–1.22)	0.313	0.447	0.75 (0.46–1.23)	0.259	0.397
Province: Qinghai	2.38 (1.18–4.81)	0.015	0.085	0.37 (0.21–0.64)	<0.001	0.007	0.64 (0.34–1.22)	0.173	0.316
Province: Heilongjiang	1.77 (1.02–3.08)	0.044	0.181	0.69 (0.43–1.09)	0.112	0.275	0.44 (0.24–0.81)	0.008	0.061
Intercept (exponentiated)	7.30 (4.60–11.59)	<0.001	—	0.08 (0.06–0.13)	<0.001	—	0.13 (0.08–0.21)	<0.001	—

Model log likelihood: I, -8680.288; C, -11427.208; P, -7693.202.

Panel C. Hospital random-intercept models restricted to secondary and tertiary hospitals

Sample: 27,088 comments from 6,571 hospitals. Outcome-positive comments: I, 24,333; C, 4,577; P, 2,324. All three models converged.

Characteristic	I OR (95% CI)	I P	I q	C OR (95% CI)	C P	C q	P OR (95% CI)	P P	P q
Sex: female (reference)	Reference	—	—	Reference	—	—	Reference	—	—
Sex: male	0.85 (0.78–0.93)	<0.001	0.002	1.12 (1.04–1.20)	0.002	0.015	1.11 (1.01–1.22)	0.033	0.120
Age: <20 years	1.02 (0.83–1.26)	0.856	0.892	0.97 (0.80–1.18)	0.794	0.864	1.03 (0.82–1.29)	0.807	0.864
Age: 20–29 years (reference)	Reference	—	—	Reference	—	—	Reference	—	—
Age: 30–39 years	1.00 (0.87–1.15)	0.999	0.999	1.12 (0.99–1.26)	0.065	0.204	0.94 (0.80–1.09)	0.393	0.522
Age: 40–49 years	0.83 (0.72–0.96)	0.014	0.071	1.42 (1.25–1.61)	<0.001	<0.001	1.14 (0.97–1.35)	0.106	0.258
Age: 50–59 years	0.79 (0.68–0.93)	0.003	0.023	1.73 (1.52–1.97)	<0.001	<0.001	1.21 (1.02–1.44)	0.026	0.103
Age: ≥60 years	0.79 (0.67–0.93)	0.004	0.025	1.73 (1.51–1.99)	<0.001	<0.001	1.04 (0.86–1.25)	0.667	0.770
Education: junior secondary or below (reference)	Reference	—	—	Reference	—	—	Reference	—	—

Characteristic	I<br>OR (95% CI)	I<br>P	I<br>q	C<br>OR (95% CI)	C<br>P	C<br>q	P<br>OR (95% CI)	P<br>P	P<br>q
Education: senior or technical secondary	0.88 (0.79–0.98)	0.022	0.096	1.21 (1.10–1.33)	<0.001	<0.001	1.20 (1.07–1.36)	0.003	0.021
Education: college or undergraduate	0.78 (0.69–0.88)	<0.001	<0.001	1.77 (1.61–1.96)	<0.001	<0.001	1.13 (0.99–1.29)	0.068	0.205
Education: postgraduate	0.65 (0.49–0.86)	0.003	0.021	2.27 (1.81–2.84)	<0.001	<0.001	1.07 (0.77–1.47)	0.695	0.783
Payment: self-pay (reference)	Reference	—	—	Reference	—	—	Reference	—	—
Payment: publicly funded medical care	0.97 (0.77–1.23)	0.812	0.864	1.05 (0.86–1.27)	0.630	0.750	0.97 (0.75–1.25)	0.807	0.864
Payment: urban employee/resident basic insurance	0.96 (0.85–1.09)	0.509	0.646	1.15 (1.03–1.28)	0.011	0.061	0.98 (0.86–1.12)	0.805	0.864
Payment: New Rural Cooperative Medical Scheme	1.02 (0.90–1.16)	0.761	0.839	1.00 (0.89–1.12)	0.992	0.999	1.01 (0.88–1.17)	0.836	0.877
Care setting: outpatient (reference)	Reference	—	—	Reference	—	—	Reference	—	—
Care setting: inpatient	1.23 (1.12–1.34)	<0.001	<0.001	0.98 (0.91–1.06)	0.660	0.767	0.55 (0.49–0.61)	<0.001	<0.001
Hospital tier: non-tertiary (reference)	Reference	—	—	Reference	—	—	Reference	—	—
Hospital tier: tertiary	1.05 (0.94–1.17)	0.383	0.513	1.15 (1.04–1.26)	0.004	0.026	0.99 (0.87–1.12)	0.824	0.870
Hospital type: general (reference)	Reference	—	—	Reference	—	—	Reference	—	—
Hospital type: traditional Chinese medicine	0.84 (0.73–0.96)	0.012	0.064	1.19 (1.06–1.34)	0.004	0.025	0.95 (0.81–1.11)	0.499	0.639
Hospital type: maternal and child health	1.01 (0.85–1.20)	0.912	0.943	0.83 (0.71–0.97)	0.016	0.077	1.09 (0.91–1.32)	0.335	0.479
Hospital type: specialised or other	0.92 (0.76–1.11)	0.383	0.513	1.21 (1.03–1.41)	0.017	0.077	0.88 (0.70–1.09)	0.231	0.385
Hospital type: integrated Chinese and Western medicine	0.62 (0.45–0.86)	0.005	0.027	1.35 (1.01–1.80)	0.042	0.146	1.29 (0.88–1.90)	0.188	0.362
Hospital type: ethnic medicine	0.48 (0.33–0.69)	<0.001	<0.001	1.82 (1.29–2.56)	<0.001	0.006	1.02 (0.63–1.66)	0.932	0.958
Comment length: per Chinese character	1.006 (1.004–1.008)	<0.001	<0.001	1.018 (1.016–1.019)	<0.001	<0.001	1.010 (1.008–1.011)	<0.001	<0.001
Province: Shanghai (reference)	Reference	—	—	Reference	—	—	Reference	—	—
Province: Yunnan	1.57 (0.96–2.57)	0.071	0.205	0.59 (0.39–0.89)	0.012	0.064	0.56 (0.32–0.97)	0.039	0.138
Province: Inner Mongolia	1.41 (0.84–2.38)	0.193	0.366	0.69 (0.45–1.08)	0.104	0.258	0.62 (0.35–1.13)	0.118	0.263
Province: Beijing	1.14 (0.66–1.95)	0.639	0.755	1.00 (0.64–1.55)	0.988	0.999	0.60 (0.32–1.11)	0.102	0.258
Province: Jilin	1.43 (0.77–2.67)	0.263	0.419	0.72 (0.43–1.20)	0.209	0.370	0.82 (0.42–1.61)	0.574	0.704
Province: Sichuan	1.48 (0.91–2.40)	0.117	0.263	0.70 (0.46–1.05)	0.083	0.225	0.65 (0.38–1.12)	0.119	0.263
Province: Tianjin	1.19 (0.59–2.38)	0.623	0.747	1.11 (0.64–1.94)	0.710	0.794	0.60 (0.26–1.37)	0.225	0.379
Province: Ningxia	1.47 (0.72–2.98)	0.286	0.438	0.72 (0.39–1.31)	0.278	0.431	0.67 (0.31–1.47)	0.320	0.466
Province: Anhui	1.40 (0.81–2.42)	0.235	0.387	0.59 (0.37–0.94)	0.027	0.103	0.89 (0.49–1.61)	0.689	0.783
Province: Shandong	1.58 (0.96–2.61)	0.073	0.207	0.77 (0.51–1.16)	0.207	0.370	0.76 (0.44–1.32)	0.331	0.477
Province: Shanxi	1.68 (0.96–2.93)	0.069	0.205	0.50 (0.31–0.81)	0.005	0.027	0.65 (0.35–1.19)	0.163	0.318
Province: Guangdong	1.41 (0.88–2.27)	0.152	0.313	0.73 (0.49–1.08)	0.117	0.263	0.72 (0.42–1.22)	0.217	0.379
Province: Guangxi	1.38 (0.83–2.29)	0.209	0.370	0.66 (0.43–1.01)	0.057	0.185	0.81 (0.46–1.41)	0.456	0.595

Characteristic	I OR (95% CI)	I P	I q	C OR (95% CI)	C P	C q	P OR (95% CI)	P P	P q
Province: Xinjiang	1.28 (0.77–2.14)	0.345	0.479	0.83 (0.54–1.28)	0.403	0.530	0.72 (0.41–1.29)	0.272	0.426
Province: Jiangsu	1.64 (0.96–2.79)	0.071	0.205	0.76 (0.49–1.18)	0.223	0.379	0.66 (0.36–1.18)	0.163	0.318
Province: Jiangxi	1.37 (0.81–2.31)	0.244	0.397	0.89 (0.57–1.38)	0.606	0.733	0.88 (0.49–1.56)	0.650	0.761
Province: Hebei	1.55 (0.94–2.57)	0.088	0.229	0.79 (0.52–1.20)	0.266	0.420	0.66 (0.38–1.15)	0.144	0.304
Province: Henan	1.39 (0.84–2.29)	0.195	0.366	0.69 (0.45–1.05)	0.083	0.225	0.78 (0.45–1.36)	0.380	0.513
Province: Zhejiang	1.16 (0.70–1.94)	0.564	0.704	0.86 (0.56–1.32)	0.483	0.624	0.76 (0.43–1.36)	0.356	0.490
Province: Hainan	1.96 (0.96–4.00)	0.065	0.204	0.31 (0.16–0.61)	<0.001	0.006	0.81 (0.38–1.71)	0.572	0.704
Province: Hubei	1.32 (0.78–2.25)	0.301	0.452	0.73 (0.46–1.14)	0.163	0.318	0.89 (0.49–1.59)	0.688	0.783
Province: Hunan	1.49 (0.88–2.53)	0.137	0.294	0.65 (0.41–1.01)	0.056	0.185	0.64 (0.36–1.15)	0.133	0.289
Province: Gansu	1.29 (0.79–2.12)	0.312	0.458	0.76 (0.50–1.16)	0.210	0.370	0.74 (0.42–1.29)	0.292	0.442
Province: Fujian	1.42 (0.81–2.51)	0.221	0.379	0.66 (0.41–1.06)	0.088	0.229	0.84 (0.45–1.56)	0.577	0.704
Province: Tibet	0.98 (0.50–1.95)	0.964	0.983	0.60 (0.31–1.13)	0.115	0.263	0.54 (0.22–1.28)	0.160	0.318
Province: Guizhou	1.54 (0.91–2.60)	0.107	0.258	0.58 (0.37–0.91)	0.017	0.077	0.85 (0.48–1.50)	0.574	0.704
Province: Liaoning	1.61 (0.93–2.78)	0.088	0.229	0.60 (0.38–0.94)	0.027	0.103	0.90 (0.50–1.62)	0.720	0.800
Province: Chongqing	1.40 (0.79–2.48)	0.246	0.397	0.80 (0.49–1.28)	0.345	0.479	0.59 (0.31–1.12)	0.108	0.258
Province: Shaanxi	1.80 (1.06–3.07)	0.030	0.113	0.80 (0.52–1.23)	0.307	0.456	0.76 (0.42–1.35)	0.342	0.479
Province: Qinghai	2.03 (1.09–3.79)	0.027	0.103	0.41 (0.24–0.72)	0.002	0.014	0.65 (0.33–1.28)	0.209	0.370
Province: Heilongjiang	1.85 (1.01–3.40)	0.047	0.161	0.70 (0.43–1.14)	0.147	0.305	0.43 (0.22–0.86)	0.017	0.077
Intercept (exponentiated)	9.23 (5.70–14.95)	<0.001	—	0.07 (0.04–0.10)	<0.001	—	0.09 (0.05–0.15)	<0.001	—

Model log likelihood: I, -8559.207; C, -11194.017; P, -7475.125.

Hospital random-intercept variance (95% CI): I, 0.429 (0.332–0.554); C, 0.425 (0.351–0.515); P, 0.863 (0.715–1.043).

Intraclass correlation coefficient (95% CI): I, 0.115 (0.092–0.144); C, 0.114 (0.096–0.135); P, 0.208 (0.178–0.241).

Panel D. Single-level logistic models with hospital-clustered standard errors restricted to secondary and tertiary hospitals

Sample: 27,088 comments from 6,571 hospitals. Outcome-positive comments: I, 24,333; C, 4,577; P, 2,324. All three models converged.

Characteristic	I OR (95% CI)	I P	I q	C OR (95% CI)	C P	C q	P OR (95% CI)	P P	P q
Sex: female (reference)	Reference	—	—	Reference	—	—	Reference	—	—
Sex: male	0.85 (0.75–0.96)	0.007	0.052	1.14 (1.06–1.23)	<0.001	0.009	1.09 (0.97–1.21)	0.141	0.298

Characteristic	I<br>OR (95% CI)	I<br>P	I<br>q	C<br>OR (95% CI)	C<br>P	C<br>q	P<br>OR (95% CI)	P<br>P	P<br>q
Age: <20 years	1.05 (0.80–1.37)	0.731	0.830	0.98 (0.82–1.18)	0.849	0.926	1.00 (0.80–1.27)	0.968	0.975
Age: 20–29 years (reference)	Reference	—	—	Reference	—	—	Reference	—	—
Age: 30–39 years	1.00 (0.84–1.18)	0.975	0.975	1.13 (0.99–1.29)	0.073	0.223	0.93 (0.79–1.09)	0.359	0.485
Age: 40–49 years	0.85 (0.71–1.00)	0.056	0.210	1.40 (1.23–1.58)	<0.001	<0.001	1.12 (0.95–1.33)	0.176	0.318
Age: 50–59 years	0.81 (0.65–1.00)	0.045	0.182	1.69 (1.43–1.99)	<0.001	<0.001	1.16 (0.93–1.44)	0.178	0.319
Age: ≥60 years	0.78 (0.65–0.94)	0.009	0.063	1.71 (1.44–2.02)	<0.001	<0.001	1.01 (0.84–1.20)	0.938	0.964
Education: junior secondary or below (reference)	Reference	—	—	Reference	—	—	Reference	—	—
Education: senior or technical secondary	0.95 (0.85–1.06)	0.373	0.499	1.15 (1.04–1.26)	0.005	0.042	1.16 (1.01–1.32)	0.032	0.145
Education: college or undergraduate	0.88 (0.76–1.01)	0.059	0.212	1.61 (1.43–1.81)	<0.001	<0.001	1.08 (0.92–1.26)	0.343	0.481
Education: postgraduate	0.72 (0.54–0.97)	0.032	0.145	2.07 (1.64–2.62)	<0.001	<0.001	0.97 (0.70–1.34)	0.851	0.926
Payment: self-pay (reference)	Reference	—	—	Reference	—	—	Reference	—	—
Payment: publicly funded medical care	0.94 (0.75–1.19)	0.620	0.756	1.10 (0.89–1.37)	0.359	0.485	1.01 (0.79–1.30)	0.926	0.962
Payment: urban employee/resident basic insurance	0.91 (0.80–1.03)	0.138	0.295	1.19 (1.06–1.34)	0.004	0.042	1.01 (0.86–1.19)	0.858	0.926
Payment: New Rural Cooperative Medical Scheme	1.04 (0.91–1.19)	0.570	0.713	0.99 (0.87–1.13)	0.930	0.962	1.01 (0.86–1.18)	0.908	0.959
Care setting: outpatient (reference)	Reference	—	—	Reference	—	—	Reference	—	—
Care setting: inpatient	1.23 (1.07–1.41)	0.004	0.042	0.98 (0.88–1.10)	0.770	0.862	0.53 (0.47–0.61)	<0.001	<0.001
Hospital tier: non-tertiary (reference)	Reference	—	—	Reference	—	—	Reference	—	—
Hospital tier: tertiary	1.10 (0.98–1.23)	0.114	0.275	1.08 (0.95–1.22)	0.257	0.397	1.00 (0.85–1.17)	0.969	0.975
Hospital type: general (reference)	Reference	—	—	Reference	—	—	Reference	—	—
Hospital type: traditional Chinese medicine	0.83 (0.71–0.96)	0.012	0.078	1.12 (0.98–1.28)	0.100	0.254	0.96 (0.81–1.13)	0.623	0.756
Hospital type: maternal and child health	1.28 (0.91–1.80)	0.161	0.315	0.69 (0.53–0.89)	0.005	0.042	1.01 (0.83–1.25)	0.890	0.947
Hospital type: specialised or other	1.01 (0.80–1.27)	0.966	0.975	1.10 (0.92–1.31)	0.294	0.425	0.90 (0.70–1.15)	0.381	0.506
Hospital type: integrated Chinese and Western medicine	0.67 (0.50–0.89)	0.006	0.051	1.24 (0.96–1.60)	0.101	0.254	1.26 (0.91–1.73)	0.163	0.315
Hospital type: ethnic medicine	0.38 (0.17–0.83)	0.015	0.085	2.31 (1.09–4.90)	0.028	0.137	0.97 (0.55–1.72)	0.930	0.962
Comment length: per Chinese character	1.006 (1.002–1.009)	<0.001	0.009	1.017 (1.015–1.019)	<0.001	<0.001	1.009 (1.007–1.010)	<0.001	<0.001
Province: Shanghai (reference)	Reference	—	—	Reference	—	—	Reference	—	—
Province: Yunnan	1.51 (0.97–2.37)	0.071	0.221	0.59 (0.39–0.88)	0.011	0.070	0.59 (0.36–0.96)	0.034	0.151
Province: Inner Mongolia	1.48 (0.89–2.46)	0.130	0.290	0.68 (0.44–1.06)	0.088	0.244	0.61 (0.36–1.02)	0.061	0.212
Province: Beijing	1.37 (0.71–2.67)	0.350	0.484	0.89 (0.56–1.41)	0.627	0.756	0.52 (0.28–0.97)	0.040	0.173
Province: Jilin	1.40 (0.80–2.45)	0.245	0.391	0.71 (0.44–1.15)	0.167	0.315	0.88 (0.45–1.73)	0.712	0.826
Province: Sichuan	1.41 (0.88–2.26)	0.150	0.302	0.70 (0.46–1.05)	0.087	0.244	0.69 (0.41–1.17)	0.167	0.315

Characteristic	I<br>OR (95% CI)	I<br>P	I<br>q	C<br>OR (95% CI)	C<br>P	C<br>q	P<br>OR (95% CI)	P<br>P	P<br>q
Province: Tianjin	1.17 (0.61–2.24)	0.630	0.756	1.05 (0.62–1.78)	0.853	0.926	0.56 (0.28–1.11)	0.097	0.254
Province: Ningxia	1.47 (0.80–2.70)	0.219	0.362	0.71 (0.38–1.32)	0.278	0.413	0.65 (0.33–1.29)	0.222	0.362
Province: Anhui	1.37 (0.83–2.27)	0.222	0.362	0.59 (0.37–0.93)	0.024	0.126	0.85 (0.50–1.46)	0.558	0.703
Province: Shandong	1.55 (0.98–2.45)	0.062	0.212	0.75 (0.50–1.11)	0.151	0.302	0.78 (0.48–1.28)	0.327	0.463
Province: Shanxi	1.60 (0.94–2.73)	0.082	0.238	0.51 (0.33–0.79)	0.003	0.030	0.64 (0.36–1.12)	0.117	0.279
Province: Guangdong	1.35 (0.87–2.10)	0.175	0.318	0.75 (0.51–1.10)	0.143	0.298	0.74 (0.46–1.20)	0.225	0.362
Province: Guangxi	1.31 (0.83–2.06)	0.253	0.395	0.67 (0.44–1.01)	0.058	0.211	0.79 (0.49–1.29)	0.352	0.484
Province: Xinjiang	1.08 (0.61–1.91)	0.786	0.873	0.89 (0.55–1.44)	0.648	0.772	0.68 (0.41–1.12)	0.128	0.290
Province: Jiangsu	1.54 (0.96–2.49)	0.076	0.223	0.83 (0.53–1.28)	0.402	0.524	0.70 (0.36–1.35)	0.286	0.421
Province: Jiangxi	1.31 (0.79–2.18)	0.293	0.425	1.18 (0.51–2.73)	0.691	0.816	0.86 (0.49–1.53)	0.614	0.756
Province: Hebei	1.53 (0.96–2.44)	0.071	0.221	0.77 (0.52–1.14)	0.196	0.333	0.67 (0.39–1.13)	0.133	0.290
Province: Henan	1.56 (0.88–2.77)	0.132	0.290	0.62 (0.35–1.08)	0.089	0.244	0.66 (0.37–1.16)	0.146	0.299
Province: Zhejiang	0.73 (0.46–1.18)	0.201	0.340	1.20 (0.79–1.84)	0.387	0.509	0.53 (0.33–0.85)	0.009	0.062
Province: Hainan	1.88 (0.94–3.76)	0.075	0.223	0.31 (0.17–0.57)	<0.001	0.002	0.80 (0.39–1.64)	0.536	0.681
Province: Hubei	0.80 (0.44–1.46)	0.463	0.599	0.73 (0.49–1.09)	0.123	0.286	1.11 (0.62–1.99)	0.721	0.826
Province: Hunan	1.40 (0.84–2.32)	0.193	0.332	0.65 (0.42–0.99)	0.047	0.184	0.68 (0.40–1.17)	0.168	0.315
Province: Gansu	1.37 (0.86–2.17)	0.183	0.323	0.71 (0.47–1.07)	0.101	0.254	0.71 (0.42–1.18)	0.185	0.323
Province: Fujian	1.09 (0.60–2.00)	0.768	0.862	1.08 (0.46–2.55)	0.866	0.928	0.72 (0.40–1.28)	0.264	0.401
Province: Tibet	1.26 (0.64–2.47)	0.503	0.645	0.52 (0.26–1.04)	0.066	0.219	0.50 (0.21–1.21)	0.124	0.286
Province: Guizhou	1.51 (0.93–2.45)	0.094	0.251	0.58 (0.37–0.90)	0.014	0.085	1.14 (0.56–2.32)	0.719	0.826
Province: Liaoning	1.65 (0.99–2.76)	0.056	0.210	0.58 (0.37–0.93)	0.022	0.117	1.19 (0.49–2.86)	0.697	0.816
Province: Chongqing	1.37 (0.80–2.34)	0.253	0.395	0.78 (0.50–1.22)	0.275	0.412	0.58 (0.33–1.04)	0.070	0.221
Province: Shaanxi	1.73 (1.06–2.82)	0.028	0.137	0.81 (0.54–1.22)	0.312	0.446	0.75 (0.46–1.23)	0.259	0.397
Province: Qinghai	2.38 (1.18–4.81)	0.015	0.085	0.37 (0.21–0.64)	<0.001	0.007	0.64 (0.34–1.22)	0.173	0.318
Province: Heilongjiang	1.77 (1.02–3.08)	0.044	0.182	0.69 (0.43–1.09)	0.112	0.275	0.44 (0.24–0.81)	0.008	0.062
Intercept (exponentiated)	7.29 (4.59–11.58)	<0.001	—	0.08 (0.06–0.13)	<0.001	—	0.13 (0.08–0.21)	<0.001	—

Model log likelihood: I, -8679.080; C, -11423.969; P, -7692.255.

Supplementary Table S6. Performance of the iFLYTEK Spark model for preliminary topic and sentiment classification

The preliminary classifier was a customised version of the iFLYTEK Spark large language model (iFLYTEK, Hefei, China). It was developed using 4,192 patient comments annotated for topic and sentiment by two trained researchers, with disagreements adjudicated by a third expert. Comments were divided into training (70%), validation (15%) and test (15%) sets.

Task	Precision, %	Recall, %	F1 score, %
Joint topic-sentiment classification	86.95	85.80	86.03
Topic classification	88.80	87.71	87.90
Sentiment classification	95.23	96.18	95.55

Note: Values describe performance on the held-out test set. The model produced classifications for 21 topics and an additional other category. A comment was retained if it was classified as positive in at least one of the 21 topics or the additional “other” category. These metrics assess preliminary classification; Table S3 reports the separate evaluation of care-practice coding. Care-practice coding combined rules with model-assisted judgments checked against the original text. Python was used to consolidate labels and check label combinations against the decision criteria.
